# Roles of *DgBRC1* in Regulation of Lateral Branching in Chrysanthemum (*Dendranthema ×grandiflora* cv. Jinba)

**DOI:** 10.1371/journal.pone.0061717

**Published:** 2013-04-17

**Authors:** Xiaoli Chen, Xiaoyang Zhou, Lin Xi, Junxiang Li, Ruiyan Zhao, Nan Ma, Liangjun Zhao

**Affiliations:** Department of Ornamental Horticulture and Landscape Architecture, China Agricultural University, Beijing, China; University of Nottingham, United Kingdom

## Abstract

The diverse plasticity of plant architecture is largely determined by shoot branching. Shoot branching is an event regulated by multiple environmental, developmental and hormonal stimuli through triggering lateral bud response. After perceiving these signals, the lateral buds will respond and make a decision on whether to grow out. TCP transcriptional factors, *BRC1/TB1/FC1*, were previously proven to be involved in local inhibition of shoot branching in Arabidopsis, pea, tomato, maize and rice. To investigate the function of *BRC1*, we isolated the *BRC1* homolog from chrysanthemum. There were two transcripts of *DgBRC1* coming from two alleles in one locus, both of which complemented the multiple branches phenotype of Arabidopsis *brc1-1*, indicating that both are functionally conserved. *DgBRC1* was mainly expressed in dormant axillary buds, and down-regulated at the bud activation stage, and up-regulated by higher planting densities. *DgBRC1* transcripts could respond to apical auxin supply and polar auxin transport. Moreover, we found that the acropetal cytokinin stream promoted branch outgrowth whether or not apical auxin was present. Basipetal cytokinin promoted outgrowth of branches in the absence of apical auxin, while strengthening the inhibitory effects on lower buds in the presence of apical auxin. The influence of auxin and strigolactons (SLs) on the production of cytokinin was investigated, we found that auxin locally down-regulated biosynthesis of cytokinin in nodes, SLs also down-regulated the biosynthesis of cytokinin, the interactions among these phytohormones need further investigation.

## Introduction

Branching types of plants have evolved over the history of life to adapt to changing climates or environmental conditions. The two processes mainly involved in the formation of lateral branches are the initiation of axillary meristems (AM) and the outgrowth of branches. During postembryonic development, the shoot apical meristem (SAM) generates the entire aerial part of a plant body [1], which can be divided into several phytomers consisting of leaf, stem segment, and AM [2]. The AMs are initiated from the axils of leaves according to the detached [3] or *de novo* [4] meristem models; after initiation, the AMs develop into axillary buds. Whether the axillary buds remain dormant or grow out to yield branches is a key decision for branching types of plants which is determined by various environmental and developmental stimuli [5].

During the initiation of AMs, there are at least two pathways involved at different developmental phases. During the vegetative growth phase of Arabidopsis, *LAS* [6], *RAX* [7], and *ROX* [8] are required to initiate AMs. The arabidopsis mutant *supershoot* (*sps*) generates several meristems in each leaf axil, which correlates with increased levels of Z-type cytokinins [9].

Another important branching event is the outgrowth of lateral buds. The repression of lateral buds by auxin production in the main shoot is known as apical dominance [10]. Auxin transports basipetally after being synthesized in young leaves, eventually inhibiting branch outgrowth [11], [12]. However, auxin has an indirect function in this process, as auxin never enters the axils [12], [13]. There are two leading hypotheses explaining this. The second messenger hypothesis is that auxin has been shown to regulate the production of cytokinins or SLs, both of which regulate branches outgrowth in the nodes locally [14], [15], [16]. However, there are evidences that buds could be activated by the efflux auxin produced in the buds [17], [18], moreover, auxin transport capacity in main stem is assumed limited [18], eventually competition of auxin transport between apices and buds leads to apcial dominance, which is named as the auxin transportation canalization hypothesis. Both hypotheses are supported by computational model [19], [20], [21] and experimental evidences [16], [22], [23], [24].

Strigolactones (SLs) are newly defined hormones involved in the inhibition of lateral branching in several species [25], [26]. Furthermore, mutants associated with greater branch production have been shown to exhibit deficiencies in SL synthesis or signaling, including the *max* mutant in Arabidopsis [27], [28], [29], [30], *dad* mutant in petunia [31], [32], [33], *rms* mutant in pea [34], [35], [36], [37], and *dwarf* mutant in rice [38], [39]. The interactions between auxin and SLs in regulation of lateral branching are complicated, SLs may act by dampening auxin transport [18], [28], [40], or they may act downstream of auxin [16], or be independent from the status of stem auxin [41] to regulate lateral branching. SLs also interacted with auxin and cytokinin in other developmental events such as adventitious root formation [42], root-hair elongation [43] or stimulation of secondary growth [44].

In addition to auxin and SLs, cytokinin also plays a role in promoting the outgrowth of branches in nodes locally [45], [46], [47]. Since auxin and cytokinin could regulate the biosynthesis and signaling of each other, it was proposed that auxin and cytokinin maintain homeostasis during plant development [15], [48], [49]. The interaction and feedback loop within these phytohormones provide a robust balance for the whole system [41], [50], [51], [52].

After perceiving the endogenous signals or environmental stimuli, the axillary buds respond and make a decision on whether to grow. A transcription factor known in various species as *teosinte branched1* (*TB1*), *branched1* (*BRC1*), or *fine culm1* (*FC1*), contains a TCP domain and is regarded as a candidate which can act locally to prevent buds outgrowth in maize [53], rice [54], sorghum [55], tomato [56] and Arabidopsis [5], [57]. *TB1* is thought to contribute to the evolution of teosinte to maize, which resulted from a profound increase in apical dominance [53], [58]. The functional role of *TB1/BRC1/FC1* is conserved in preventing branch outgrowth in both monocots and dicots, while the TB1 in maize also plays a role in internode elongation and inflorescence development [5], [53]. Moreover, *BRC1* in pea and *FC1* in rice act downstream of the SL pathway, and their functions are essential for SL mediated inhibition of bud outgrowth [59], [60]. New evidence has proven that another class I HD-Zip transcriptional factor, *GRASSY TILLERS 1*(*GT1*), is a local regulator of tillering and consequently influences lateral branching in maize, moreover, the expression of *GT1* was under the control of *tb1* [61].

The branching pattern of ornamental plants determines their esthetic appeal, and hence, their commercial value. Chrysanthemum (*Dendranthema grandiflorum*) is one of the important standard cut flowers, and requires manual decapitation or removal of lateral branches to maintain its architecture, which comprises one-third of the production cost. We previously reported that strigolactones regulate lateral branching in the presence of auxin source in chrysanthemum [40], and that *DgIPT3* isolated from chrysanthemum engages in cytokinin biosynthesis and lateral branching [62]. Other studies provided approaches to control the lateral branching of chrysanthemum, such as the transformation of antisense *DgLsL* [63], [64]. The genetic network of branching in chrysanthemum needs further elucidation to provide breeders with new methods to cultivate novel cultivars with ideal traits. Here we build on this understanding by identifying the roles of *DgBRC1* in regulating lateral branching under endogenous and exogenous stimuli. Additional investigations describe the interactions among auxin, cytokinin, and SLs in the regulation of shoot branching.

## Results

### Apical dominance in chrysanthemum

While considering the multi-branching nature of chrysanthemum plants, the branching characteristics of chrysanthemum cultivar ‘Jinba’ grown in a greenhouse were recorded during different growth stages. We recorded the length of all buds along the whole plant when the plant heights were 45 cm, 65 cm and 85 cm, respectively. The positions of buds were numbered acropetally, where bud 1 was the lowest bud (Figure S1). All the buds on the 45 cm high plants remained dormant, and started to activate at the lower positions (around buds 21 to 26) when the plants reached 65 cm. Gradually, several active buds elongated and produced branches (around buds 19 to 27 in 85 cm high plants). Interestingly, the lowest buds usually maintained dormancy or expanded by a few leaves without further elongation during the entire growth period. Thus, the activation of axillary buds in chrysanthemum was associated with the developmental status and the distance from the shoot apex, exhibiting the same pattern observed in other species [2]. Additionally, a basipetal elongation of lateral branches was observed during the reproductive phase (Figure S2).

The classic experiment of apical dominance showed that removal of the SAM resulted in the activation of lower AMs and production of lateral branches [10]. For the assays below about decapitation or isolated stem segments, the buds/branches were numbered basipetally where bud 1 was the top bud whose attached leaf expanded to 10 mm below the shoop tip, and the bud 2 was the bud below bud 1. Following decapitation of chrysanthemum, the top three buds elongated. Fifteen days post-decapitation, buds 1 and 2 had the same growth rate and yielded 17 mm branches, whereas buds 4∼6 were activated but did not show obvious elongation due to correlative inhibition (Figure S2) [65].

### Isolation of *BRC1* homologue from chrysanthemum

**Figure 1:**
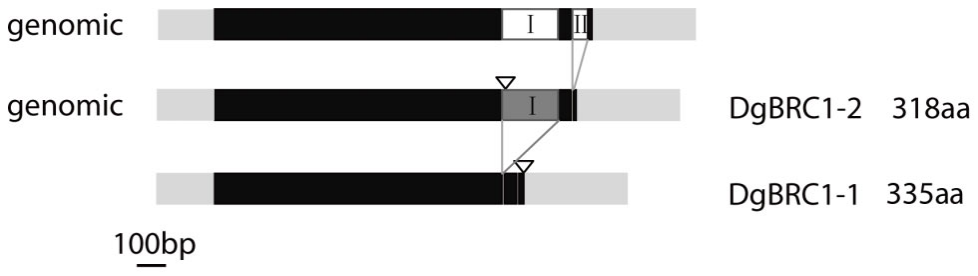
Structure of the *DgBRC1* genes. Coding sequences are shaded in black, introns in white, 5′ UTR and 3′ UTR in light grey. The two segments isolated from the cDNA were named as *DgBRC1-1* and *DgBRC1-2*. In *DgBRC1-2*, the alternative intron (intron I) is kept (indicated by dark grey box), which ended the protein later. Two copies coming from genomic DNA are shown. The termination codons are indicated by a triangle.

**Figure 2:**
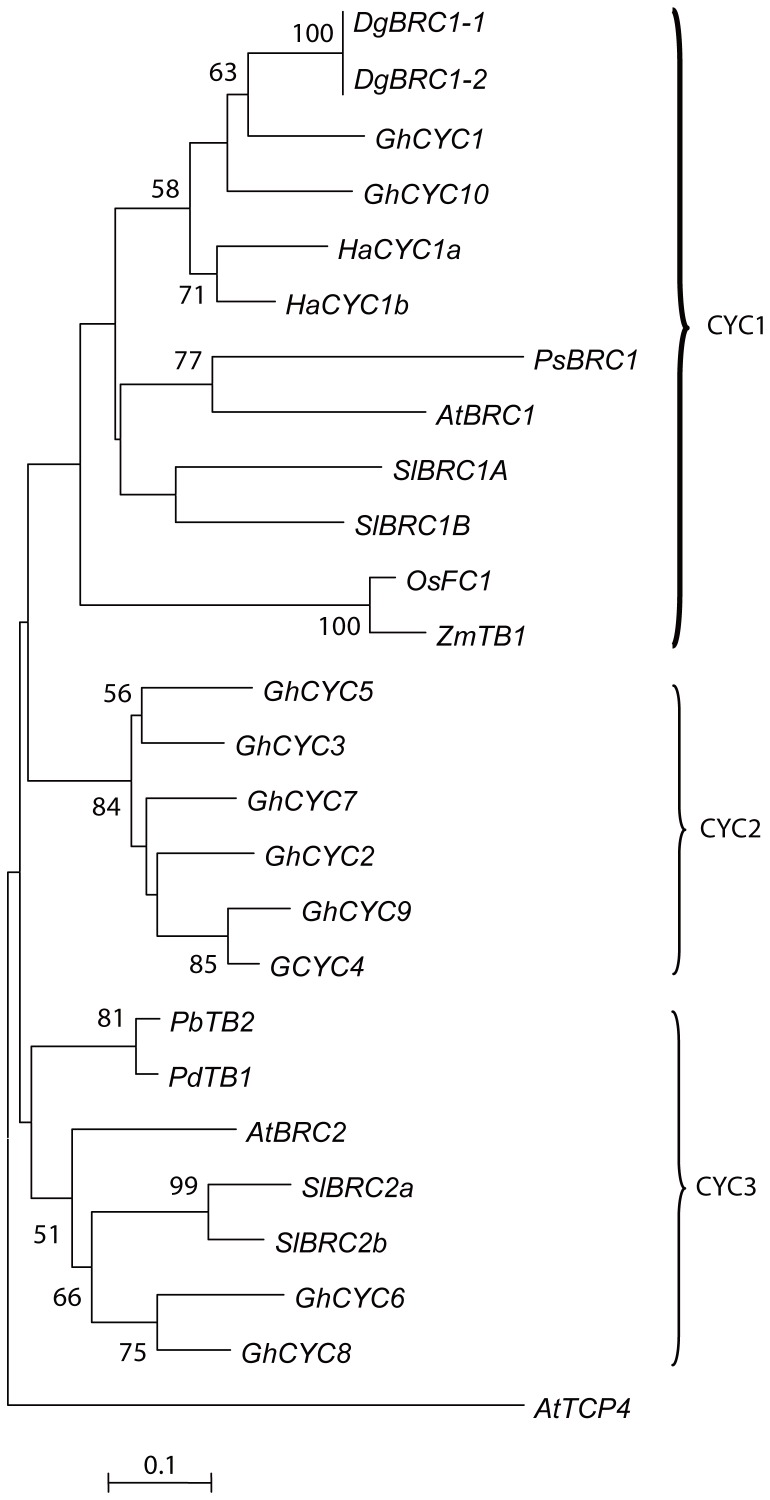
Subcellular localization of DgBRC1 alleles. DgBRC1-1-GFP (A), DgBRC1-2-GFP (B), DgBRC1-1Δ17-GFP (C) and C-terminal sequences of three proteins (D) are shown. Images A, B, C, the bright-field, GFP fluorescence, and merged images of the same onion epidermal cells are presented from left to right respectively. DgBRC1-1-GFP localized in nuclei (A), while DgBRC1-2 -GFP localized in nuclei and plasma membranes (B). DgBRC1-1Δ17-GFP accumulated in nuclei and plasma membranes in 5 of 25 cells (C), whereas the others accumulated in nuclei (data not shown). The C-terminal sequences of DgBRC1-1, DgBRC1-2, DgBRC1-1?17 are shown in D, the extra 17 amion acids in DgBRC1-1 and the mutated 17 amino acids in DgBRC1-1Δ17 are both underlined.

**Figure 3:**
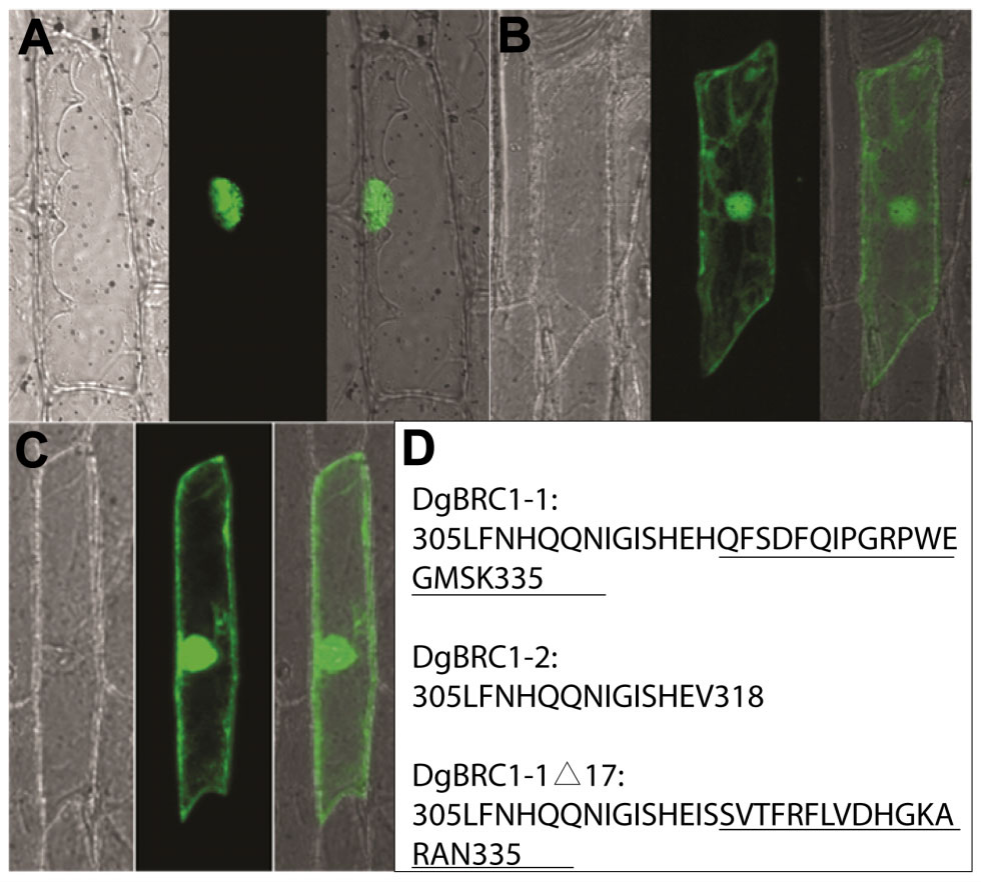
Phylogenetic tree of selected TCP proteins. Maximum likelihood (ML) phylogenetic tree was analyzed with 100 bootstrap pseudoreplicates of class II TB1/CYC genes from *Dendranthemum grandiflora* and representative class II TCP members from *Arabidopsis thaliana* (At), *Gerbera hybrid* (Gh), *Helianthus annuus* (Ha), *Pisum sativum* (Ps), *Oryza sativa* (Os), *Solanum lycopersicum* (Sl), *Populus deltoids*(Pd), *Populus balsamifera*(Pb), *Zea mays* (Zm). Branches with support of 50 or more are indicated. AtTCP4 is in an outlying group. Clades are named according to [70] [75]. Accession no. is listed in Table S1.

To study the role of *BRC1*-like transcription factors during the development of lateral branches, two cDNA clones contained the TCP domain were isolated from the axils of chrysanthemum leaves. The1362 bp clone encodes a 335 amino acid protein, and the 1545 bp clone contains an extra 183 bp segment adjacent to the 3′ UTR which introduces a stop codon, truncating the protein at 318 amino acids (Figure 1, Figure 2D); these two segments were named as *DgBRC1-1* and *DgBRC1-2*, respectively. In *DgBRC1-2*, only one amino acid is encoded in the un-spliced intron I and then a stop codon occurs (Figure 1). Both copies contain the conserved TCP and R domains, and the ECE motif typical for the CYC/TB1 clade of the TCP family (Figure S3) [5], [66]. Two segments were cloned from genomic DNA; one has the same sequence as *DgBRC1-2*, whereas the other has a 49 bp intron (intron II) (Figure 1). Because that these clones were almost identical, even in the 3′UTR region, so they were alleles from the same locus. As a hexaploid, chrysanthemum is thought to be a hybrid originated from several ancestries such as *D. vestitum*, *D. indicum* and *D. nankingense* [67], so there may be several alleles with same or different functions in the locus. We performed a phylogenetic analysis with a selected set of class II TCP factors from various plant species to explore the evolutionary relationship among *DgBRC1* genes. The nucleotides sequences coding the conserved TCP domains were aligned and maximum likelihood analyses were performed. As expected, *DgBRC1* belongs to the CYC1subclade of the CYC/TB1 clade of the class II TCP family along with other *BRC1*-like genes such as *PsBRC1*, *SlBRC1a*, *SlBRC1b*, and *AtBRC1* (Figure 3), all of which were shown to retain the function of *tb1* in regulating lateral branching [5], [56], [57], [60].

The basic helix-loop-helix (bHLH) motif of the TCP domain was predicted to promote DNA binding and protein–protein interactions [66], [68], and some TCP proteins have been shown to be targeted to the nucleus [5], [69], [70]. According to our analysis of subcellular localization, DgBRC1-1-GFP localized to nuclei, but DgBRC1-2-GFP was dispersed all over the cells (Figure 2). Compared with DgBRC1-2, DgBRC1-1 has a 17 amino acid tail; we mutated the nucleotides coding these 17 amino acids by inserting 2 nucleotides, resulting in a frameshift mutation (Figure 2D). Interestingly, DgBRC1-1Δ17-GFP still accumulated in nuclei in 20 of 25 cells observed (data not shown), and were widely dispersed in another 5 cells (Figure 2C). To conclude, the peptide in the C-terminal of DgBRC1-1 is partly necessary for nuclear localization, however, the requirements may not be sequence specific.

### *DgBRC1* is mainly expressed in dormant axillary buds

**Figure 4:**
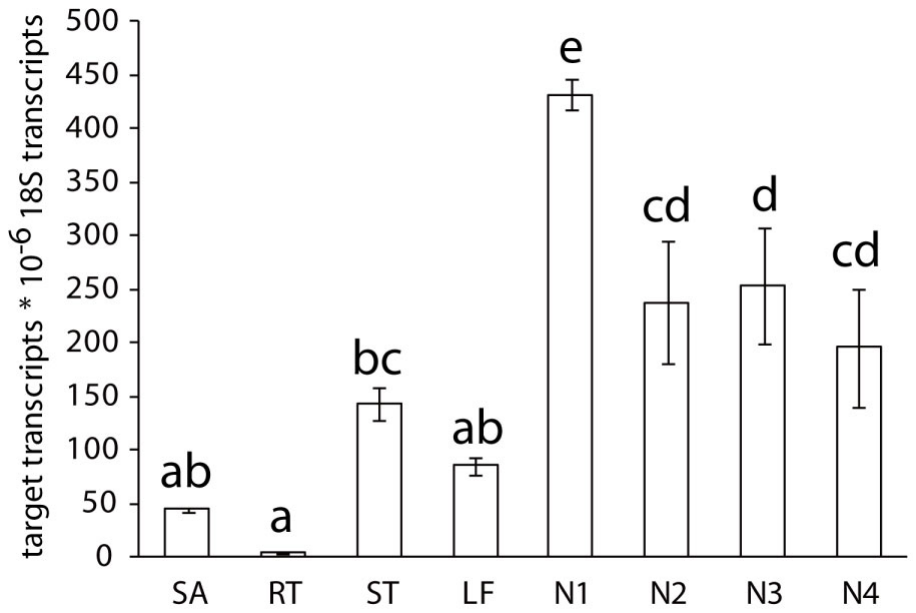
Transcript levels of *DgBRC1* in different tissues. Total transcript levels of *DgBRC1* in different tissues were analyzed by real-time PCR. Bud position was recorded basipetally. Error bars indicate SE from three biological replicates consisting of 10 plants for each replicate. Abbreviations are SA, shoot apex; RT, root; ST, stem; LF, leaf; N1, node 1; N2, node 2; N3, node 3; N4, node 4.

To investigate the expression pattern of *DgBRC1* during the vegetative phase of chrysanthemum, *DgBRC1* transcripts were quantified by Real Time PCR. *DgBRC1* was mainly expressed in the nodes containing axillary buds, which supported their roles in shoot branching (Figure 4). *DgBRC1* was weakly expressed in the stem, leaf, and main shoot, while its expression in root was barely detectable. The highest level of expression was in the first node below the main shoot, and remained high in nodes 2 to 4 (Figure 4). The *DgBRC1-1* transcripts levels were also detected in different tissues, whose expression pattern was similar to total *DgBRC1* (data not shown).

### Functional conservation of the *DgBRC1s*

**Figure 5:**
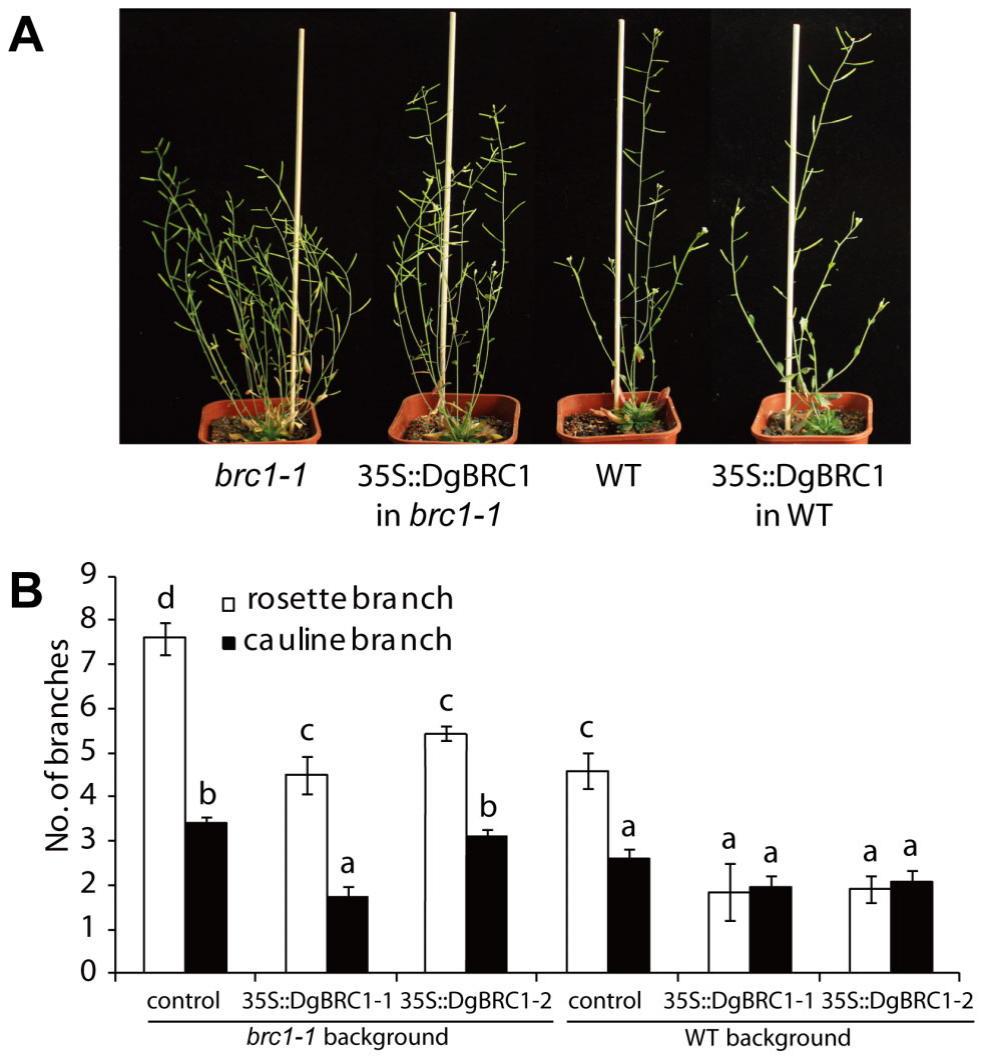
Phenotype of 35S::DgBRC1 of WT and *brc1-1* Aribidopsis plants. (A) Shoot branching phenotypes of WT and *brc1-1*with and without the 35S::DgBRC1 variant 1 construct. (B) Primary rosette and cauline branch number of WT, *brc1-1* and 35S::DgBRC1 lines. All plants were grown with long days under the same conditions and recorded at 10 days after anthesis, the number of the primary rosette and cauline branches longer than 3 mm were recorded. Data are means ± SE; n = 16. Letters indicate significant differences between them at α = 0.05.

To determine whether *DgBRC1* is functionally conserved, the *DgBRC1* ORFs were overexpressed from the Cauliflower Mosaic Virus 35S promoter (35S::DgBRC1-1, 35S::BRC1-2) in the Arabidopsis WT and *brc1-1* mutant. Multiple independent transgenic lines were generated with each construct, and those showing Medelian segregation patterns 15∶1 in T2 lines were taken to homozygosity for detailed analysis. The numbers of rosette and cauline branches with a length of at least 3 mm were scored 10 days post-anthesis (DPA). Figure 5A represents typical lines for each construct with 35S::DgBRC1-1 (35S::DgBRC1-2 lines were similar and not shown). Overexpression of *DgBRC1* reduced the number of rosette branches from 7.6 in *brc1-1* to 4.5–5.7, which was indistinguishable from wild-type (WT) plants with an average of 4.6 branches (Figure 5B, Table S2). Overexpression of *DgBRC1* in WT inhibited the total growth of WT plants, and reduced the number of rosette branches from 4.6 to 1.8–2.1 (Table S2). These results indicated that both variants retain conserved functions of regulating lateral branching.

### Accumulation of *DgBRC1* transcript is regulated by apical dominance and planting density

**Figure 6:**
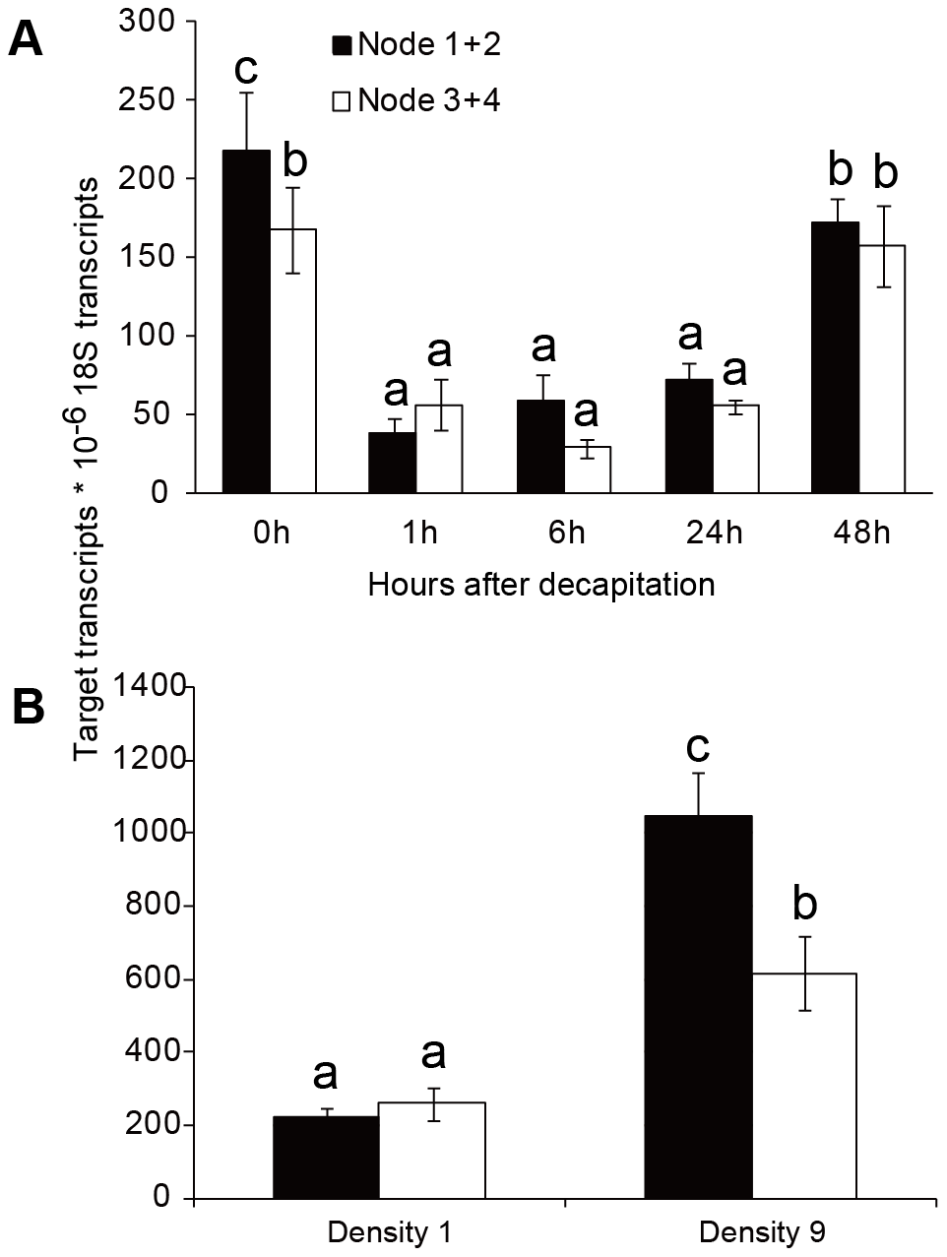
Transcript levels of *DgBRC1* after decapitation and at different planting densities. (A) *DgBRC1* transcript levels in node 1+2 and node 3+4 were analyzed 0 h, 1 h, 6 h, 24 h and 48 h after decapitation by real-time PCR. Bud position was recorded basipetally. (B) *DgBRC1* transcript levels at density 1(1 plant per 729 cm^3^) and density 9 (9 plants per 729 cm^3^). Results are means of three biological replicates with 10 plants for each replicate.

To determine the effects of apical dominance on *DgBRC1* transcript levels, the classical decapitation assay was conducted. As mentioned earlier, the top three buds were released predominantly after decapitation (Figure S2). To determine whether the outgrowth of lateral branches correlates with down-regulation of *DgBRC1*, transcript levels were analyzed in node groups 1 and 2 (node 1+2, top two nodes), and node groups 3 and 4 (node 3+4) before any visible sign of bud outgrowth. After decapitation, the transcript levels of *DgBRC1* in nodes decreased dramatically 1 h following decapitation, and then almost attained pre-decapitation levels by 48 h after decapitation (Figure 6A). We conclude from these results that *DgBRC1* transcription was down-regulated rapidly when the inhibitory effect of SAM on lateral buds was released.

The response of plants to high planting density is known as shade avoidance syndrome [71], [72], which includes a decrease of lateral branching [73]. In Arabidopsis, high planting density was found to regulate the outgrowth of branches partly through *BRC1* [5], [74]. To test whether *DgBRC1* transcript levels were sensitive to planting density, chrysanthemum seedlings were planted in 1 or 9 plants per pot (9 cm×9 cm×9 cm), respectively. No matter in nodes 1+2 or 3+4, the *DgBRC1* transcript levels in high density conditions (9 plants per pot) increased significantly compared with the lower density (1 plant per pot)(Figure 6B). These results indicated that the shade avoidance response of chrysanthemum correlates with *DgBRC1* transcript levels.

### Auxin, bud outgrowth and *DgBRC1* transcripts

**Figure 7:**
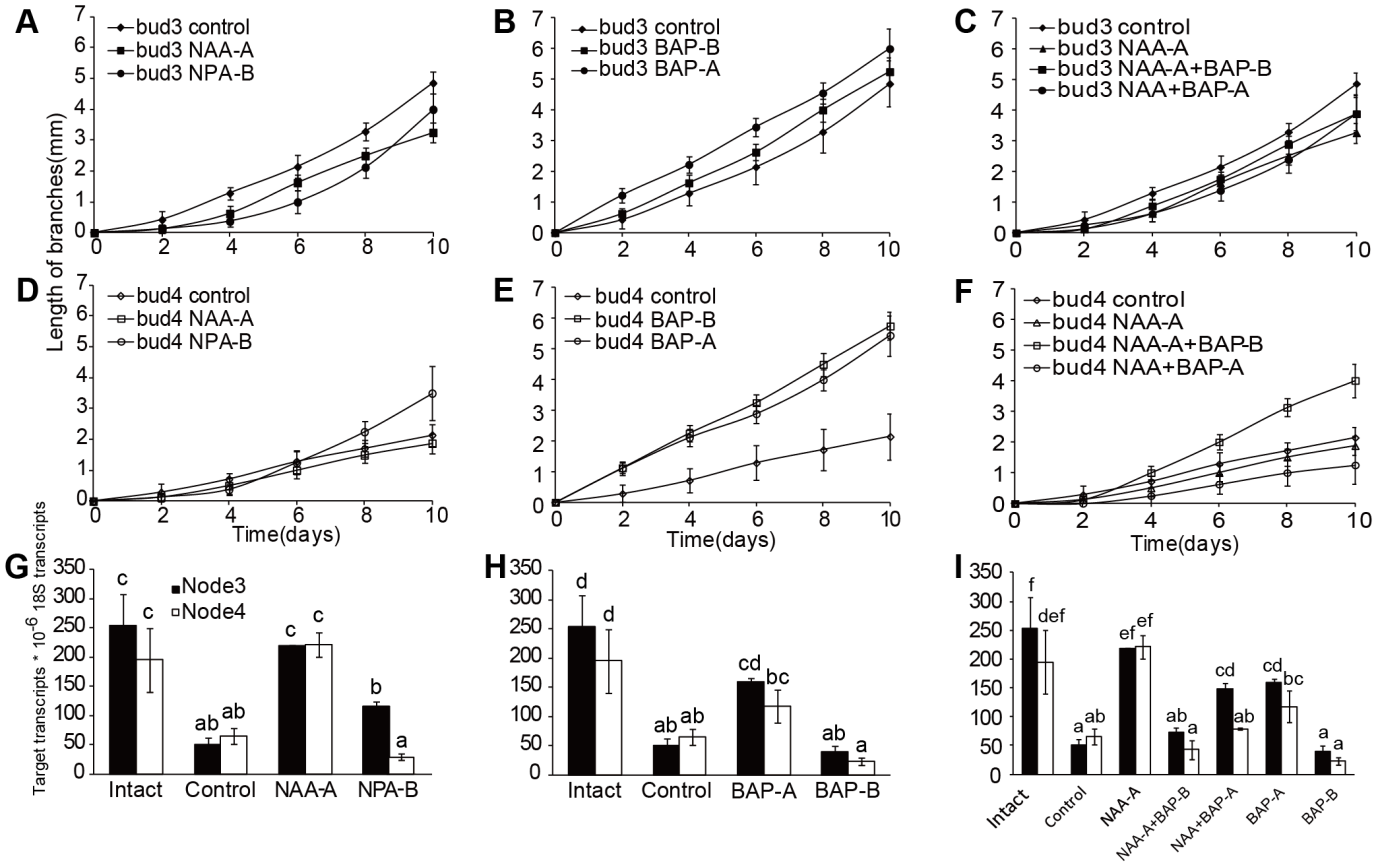
Elongation of two-bud stem segments and transcript levels of *DgBRC1* after PGR application. Bud position was recorded basipetally. Stem segments containing bud 3 and bud 4 were used as plant materials for PGR application. The elongation dynamics of bud 3 (A to C) and bud 4 (D to F) 10 days after application of PGR are presented. Figures G to I indicate the transcript levels of *DgBRC1* 4 hours after application of PGR. There were three groups of PGR treatments: left, 5 µM NAA and NPA applied; middle, 5 µM BAP applied; right, 5 µM NAA and BAP applied together. Stem segments without any PGR were served as the control. Node 3 and node 4 were collected 4 h after treatment for *DgBRC1* transcript analysis. Data were means ± SE. For lateral branches outgrowth, n = 8. For gene expression, results are means of three biological replicates analyzed by real-time PCR, with 10 plants for each replicate; letters indicate significant differences between different PGR applications at α = 0.05. PGR application: NAA on apical sides (NAA-A), NPA on basal sides (NPA-B), BAP on apical or basal sides (BAP-A, BAP-B), NAA and BAP on apical sides (NAA+BAP-A), NAA on apical sides and BAP on basal sides (NAA−A+BAP-B).

To investigate transcript levels of *DgBRC1* regulated by auxin, plant growth regulators (PGR) were applied on two-bud segments including bud 3 and bud 4 cultured in a split-plate system (Figure S4) [46]. *DgBRC1* transcript analysis was performed on both nodes 3 and 4 following 4 hours of treatment with 5 µM NAA applied apically and 5 µM NPA applied basally; the elongation of branches was recorded 10 days after treatment. In the intact plants no activation of the buds at nodes 3 or 4 was observed over the course of the experiment, so the growth of branches in intact plants was not shown in Figure 7. Compared with the two-bud segments without any PGR applied (control), 5 µM NAA in the apical media block was found to slightly inhibit the outgrowth of bud 3, and did not alter the growth of bud 4 (Figure 7A, D); the *DgBRC1* transcript levels in node 3 and node 4 were restored to the levels in buds of intact plants, which did not correlated with the observed changes in outgrowth of bud 3 and buds 4 (Figure 7G). 5 µM NPA in the basal media block had modest effects on outgrowth of branches (Figure 7A, D) and *DgBRC1* transcript levels when compared with the control (Figure 7G). Interestingly, transcription of *DgBRC1* in node 3 was higher than in node 4 after NPA application, which could be explained by the inhibition of the PATS by NPA from bud 3 to bud 4.

### Cytokinin, bud outgrowth and *DgBRC1* transcripts

To investigate the effect of cytokinin on transcripts of *DgBRC1*, 5 µM synthetic cytokinin Benzylaminopurine (BAP) was added to the apical or basal media blocks. Nodes 3 and 4 were sampled separately 4 hours after PGR treatment, and the elongation of branches were recorded 10 days thereafter. Both apically and basally applied BAP promoted elongation of both buds, especially buds near to the application position (Figure 7B, E). Basally applied BAP on two-bud sections modestly reduce the *DgBRC1* transcript levels, while apically applied BAP did not down-regulate transcription of *DgBRC1* in both nodes (Figure 7H). These results indicated that cytokinin promoted elongation of lateral branches, but it was not correlated with the transcripts of *DgBRC1*. *DgBRC1* may be regulated post transcriptionally, or there may be other pathways independent of *DgBRC1* which can respond to cytokinin.

### Antagonism between auxin and cytokinin on lateral branching

To investigate the antagonistic effects of auxin and cytokinin on outgrowth of lateral branches and transcripts of *DgBRC1* in chrysanthemum, two assays were designed. Firstly, both 5 µM NAA and BAP were supplied apically; secondly, apical 5 µM NAA and basal 5 µM BAP were supplied simultaneously. Comparing Figure 7F with 7D and 7E, basal cytokinin's promotion of growth in bud 4 was suppressed a bit by the apical auxin, but apical cytokinin's promotion of growth of bud 4 was countered by apical auxin. In another way, basal BAP weaken the inhibition by apical auxin, while apical BAP strengthened the inhibition of apical auxin on outgrowth of lateral branches. According to Figure 7I, apical applied auxin did not alter the transcripts of *DgBRC1* no matter BAP was applied apically or basally.

### Effect of Auxin and Strigolactone on *DgIPT3* transcript in nodes

**Figure 8:**
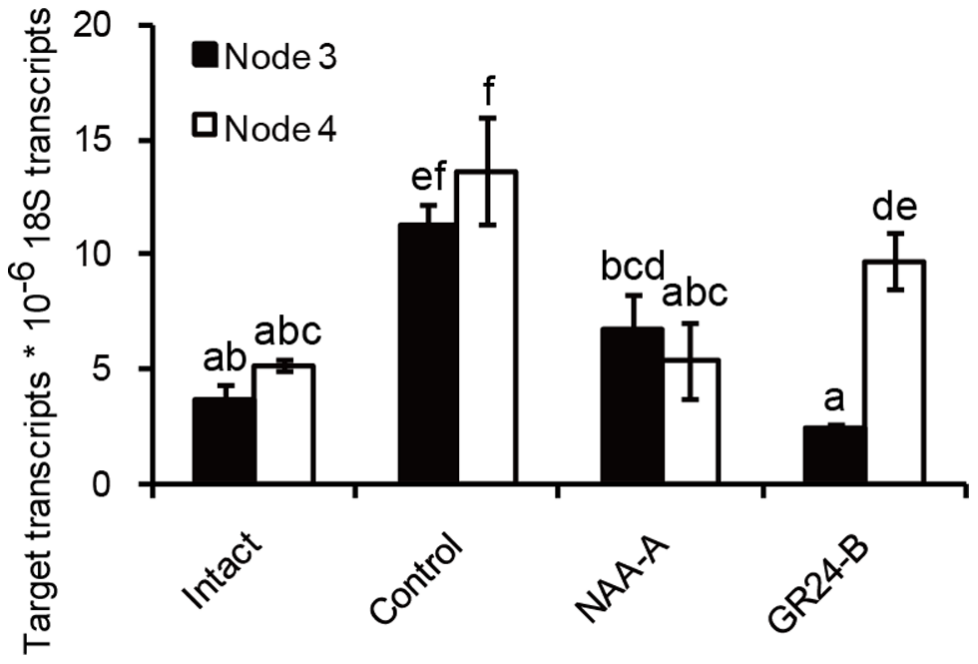
*DgIPT3* transcript patterns in nodes 3 and 4 after NAA and GR24 treatments. There were four treatments, including intact plants, control, NAA-A, GR24-B. Plant materials and experiment procedures were prepared as Figure 7. Nodes 3 and node 4 were collected 6 h after treatment for analysis of *DgIPT3* transcript levels. Total RNA was subject to quantitative real-time PCR. Results are means of quantitative PCR analyses from three biological replicates, with 10 plants for each replicate; letters indicate significant differences between treatments at α = 0.05. PGRs applied in assays: NAA on apical sides (NAA-A), GR24 on basal sides (GR24-B).

To test the effects of auxin and strigolactone on biosynthesis of cytokinin, transcript levels of *DgIPT3* in nodes were investigated 6 hours after application of apical NAA, basal GR24 (a synthetic SL). According to Figure 8, the *DgIPT3* transcript levels in two-bud segments (control, as decapitated plants) were enhanced significantly compared with those from intact plant, while *DgIPT3* transcript levels were decreased to the level of intact plants when apical NAA was applied (Figure 8). Basal GR24 reduced the *DgIPT3* transcript levels compared with the control, especially for node 3, which had *DgIPT3* transcript levels significantly different from those of node 4.

To conclude, apical auxin could reduce *DgIPT3* transcript levels of in the nodes, implying that auxin reduces the biosynthesis of cytokinin in axillary buds locally. In addition, SLs could also reduce *DgIPT3* transcript levels in the nodes.

## Discussion

### Structure of *DgBRC1* genes

The *DgBRC1* genes described in this study possessed all the characteristics of TCP family members in other species, such as the TCP domain, R domain, and especially the ECE motif which is contained in the CYC/TB1 clade [75]. TCP genes containing a bHLH motif have been shown to be involved in regulation of plant growth and development [66], [68], [76]. Two subfamilies of TCP proteins are further subcategorized into class I [68], and class II [53], [77], [78] based on phylogenetic analyses. The class II TCP proteins could be further divided into two groups: the CINCINNATA (CIN) clade [79] and CYC/TB1clade [5], [80]. In core eudicots, CYC/TB1 has been duplicated twice, generating three subclades: CYC1, CYC2, and CYC3 [75]. The CYC1 subclade mainly functions to regulate lateral branching [5], [56], [57]. The CYC2 subclade plays a role in determination of flower symmetry [80], [81], [82]. The function of CYC3 subclade is poorly understood, the *BRC2* gene from Arabidopsis played a minor role in the outgrowth of lateral branches [5], and may be involved in coordination of growth among branches [74].

Interestingly, *DgBRC1* also shares some rare characteristics with other members in this family, such as introns located within 3′ UTRs, which has previously been reported for *CYC* in Antirrhinum [82], and three *CYC2* genes in sunflower [83]. Introns in the 3′UTR may engage in nonsense-mediated mRNA decay (NMD), which could identify and eliminate aberrant mRNAs or regulate gene transcription [84], [85].

The TCP domain is considered necessary but not sufficient for DNA binding and protein-protein interactions [5], [68], [69], [70], [86]. In our assay, two alleles were found to differ in subcellular localization; the short one localized to the cytoplasm and nuclei, whereas the long one displayed nuclear localization. Most cells transformed by the mutated DgBRC1-1 in the extra 17 amino acids of the C-terminal still localized in the nuclei. These results indicated that the extra 17 amino acids were necessary for nuclear localization, but the sequence may be not specific.

### Basipetal and acropetal cytokinin regulated lateral branching in different pathways

There is evidence that xylem cytokinins (mainly tZR type) are transported through the transpiration flow acropetally as long-distance signals, and phloem cytokinins (mainly iP type) are translocated either systemically or basipetally [87]. Xylem cytokinins were supposed to promote sustained outgrowth of lateral branches [21]. In our work, cytokinins supplied in apical or basal medium were supposed to transport in different directions. Both basipetal and acropetal cytokinin promoted the outgrowth of buds 4 when apical auxin was absent, seemed that the correlative inhibition was released. However, basipetal cytokinin strengthened the inhibition on lower buds manipulated by apical NAA, which was similar to results in other plants [46], [88], [89], [90], [91], [92], while acropetal cytokinin weakened the repression of auxin on buds outgrowth. Basipetal and acropetal cytokinin also played different roles in regulation of *DgBRC1* transcripts in short term. In consequence, cytokinins from different directions or sources may be involved in different pathways.

### Roles of *DgBRC1* in regulation of lateral branches

*BRC1* loss-of-function mutants yielded multiple branches in various plant species [5], [53], [54], [56], [57], [60], and transcripts of *BRC1* were down-regulated upon release of apical dominance; furthermore, *BRC1* has been shown to respond to other hormonal or environmental stimuli [5], [57]. A strong correlation between bud repression and expression of *BRC1* genes has been proposed [93]; therefore, *BRC1* was thought to be an integrator of branching signals which could control the outgrowth of lateral branches [5].

In our work, *DgBRC1* transcripts responded rapidly to the release of apical dominance by decapitation, in addition, 4 hours after auxin was applied to the stem segments apically, transcripts level of *DgBRC1* returned to the level of intact plants. These results indicated that *DgBRC1* transcripts were related with auxin regulation of buds outgrowth in short term. In another aspect, *DgBRC1* transcripts were higher in high planting density, which indicated that *DgBRC1* was also related with shade avoidance syndrome. However, *DgBRC1* transcripts could not respond to cytokinin applied apically or basally at least in short term. Results from other studies suggest that, more branches were generated when pea *brc1* mutants were decapitated or supplied with BAP, which indicated other *BRC1*-independent pathways could control the outgrowth of branches [60]. Furthermore, transcripts of *FC1* remained high in active buds of a rice strigolactone mutant [38], and *SbTB1* transcripts did not increase when strong buds arrest was caused by defoliation [94]. In conclusion, *BRC1* may be not sufficient or necessary for outgrowth of lateral branches.

### Auxin regulates cytokinin biosynthesis in nodes locally

Concentration of cytokinin in xylem exudates or axillary buds increased after decapitation [48], [95], [96], [97], and auxin could regulate the biosynthesis of cytokinin, which makes cytokinin a good candidate as the second messenger of auxin [15], [48]. Two hypotheses have been proposed regarding the sites at which auxin effects cytokinin biosynthesis. Since root is the main place of cytokinin synthesis, it was supposed that auxin effects biosynthesis of cytokinin in root, followed by cytokinin transport into axillary buds through xylem. To support this, transcripts of *BrIPT3* and *7* in root were reduced by NAA incubation [98]; the biosynthesis of cytokinin was iPMP dependent in roots of Arabidopsis and tobacco, which could be down-regulated by auxin [15]. However, there is other evidence suggesting that the cytokinin promoting axillary bud outgrowth after decapitation was locally biosynthesized in the nodal stems rather than in the roots [48]. After decapitation, *PsIPT1* and *2* transcripts of in nodal stems increased dramatically, but transcripts in axillary buds remained unaffected [48]. In rice, expression of *OsIPT2, 4, 7* and *8* in the shoot apex (containing the SAM, axillary buds, young leaves and nodes) were clearly suppressed within 3 h following auxin treatment [59]. Here we showed that *DgIPT3* transcripts in nodes were up-regulated after decapitation, and dropped back to the levels of intact plants when auxin was applied apically on decapitated plants, which indicates that auxin could regulate the biosynthesis of cytokinin in nodes locally.

### The interactions among auxin, cytokinin, and SLs

Since the mutants of SLs production and signaling were investigated and then the characteristic of SLs was revealed [25], [26], SLs appear to be involved in optimizing several plants growing and developmental events in lower and higher plants [42], [43], [44], [99]. During these events, SLs were found to be interacted with other hormone to balance the homeostasis of plants [42], [43], [44], [99]. In the events of lateral branching, the interactions between SLs and auxin were complicated, for example, SLs was supposed to regulate shoot branching by dampening auxin transport, which is supported by direct evidence that GR24 inhibited branching only in the presence of auxin in the main stem in Arabidopsis and chrysanthemum [23], [40], and PIN1 accumulation in xylem parenchyma cells was reduced by GR24 [18], [23]; other evidences indicated that, SLs acted as a second messenger to repress shoot branching [16], and SLs could repress lateral branching directly without requirement of apical auxin [16], [99].

Auxin and cytokinin were shown to play antagonistic roles in regulation of shoot branching. Auxin could inhibit the biosynthesis of cytokinin in root [15], [95] or stem [48], in our work auxin inhibited cytokinin biosynthesis in nodes, which was supposed to be more fast and effective to regulate lateral branching. Furthermore, cytokinin could regulate the biosynthesis of auxin [50], and phloem cytokinin could modulate the polar transport of auxin [100]. A homeostasis feedback loop via auxin and cytokinin biosynthesis, transports, and signaling is thought to regulate several important developmental events [46], [90].

There has been no direct evidence about the interactions between SLs and cytokinin. In our study, when GR24 was applied basally in stem segments, the *DgIPT3* transcripts levels were reduced in nodes compared with stem segments without any hormone supplied, which suggested that SLs may inhibit the biosynthesis of cytokinin. Interestingly, *DgIPT3* transcripts level in node 3 was much lower than node 4, which may be explained as that SLs dampened the transport of auxin from node 3, and the accumulation of auxin further repressed the biosynthesis of cytokinin. In addition, auxin could inhibit the biosynthesis of SLs in stems [101], so it is equally possible that auxin induces SLs to repress cytokinins biosynthesis in our work. Therefore, auxin and SLs could be in a dynamic feedback loop to regulate cytokinin biosynthesis and to control lateral branching. From the above, auxin, cytokinin and SLs are in a dynamic feedback loop, and further studies are needed to reveal this network in regulation of branching.

## Materials and Methods

### Plant materials and growth condition

Chrysanthemum (*Dendranthema grandiflorum* cv. Jinba) plantlets were propagated under sterile conditions in jars containing MS agar medium [102], and then grown in tissue culture room at 24°C with a photoperiod of 16/8 h light/dark and a light intensity of 100–120 µmol m^−2^ s^−2^. For the apical dominance characterization assay, plants were transferred into pots (9 cm×9 cm×9 cm) containing peat soil and vermiculite (1∶1) in a green house at 24°C, with a photoperiod of 16/8 h light/dark. For the planting density assay, seedlings were cultured with 1 plant per pot, or 9 plants per pot. Seeds of *Arabidopsis thaliana* Col-0 (WT) and *brc1-1* were stratified for 3 d at 4°C, and then sown in cells containing peat soil and vermiculite (1∶1); they were transferred to a chamber at 22°C with a photoperiod of 16/8 h light/dark.

### RNA extraction and gene isolation

Total RNA was extracted from nodes with TRIzol Reagent (Invitrogen, 15596-026); cDNA synthesis was performed using SuperScript II reverse transcriptase (Invitrogen, 18064-022). Chrysanthemum TCP domain factors were amplified from cDNA prepared from nodes using PCR with two different pairs of degenerate primers (Table S3). After amplification of a fragment containing a partially conserved TCP and R domain, full-length cDNA of *DgBRC1* was elongated by 5′and 3′ Rapid Amplification of cDNA Ends (RACE) PCR. Complete *DgBRC1s* from both cDNA and genomic DNA were cloned with primers specific to the 5′UTR and 3′UTR. Amplified fragments were cloned into the pMD18-T vector (Takara, D101A) and sequenced. Genomic DNA was extracted from young leaves using the CTAB method.

### Sequence Alignment and Phylogenetic analyses

DNA Sequences from the start of the TCP domain to the end of the R domain were aligned with ClustalW2 (http://www.ebi.ac.uk/Tools/msa/clustalw2/) using default parameters [103], and visualized with Genedoc [104]. During phylogenetic analyses, TCP domain-coding DNA sequences were aligned with MUSCLE (http://www.ebi.ac.uk/Tools/msa/muscle/) using default parameters [103]. The test for best nucleotide substitution evolutionary model was done with jMODELTEST [105], [106]. The best fit model (Akaike Information Criteria selection) was GTR+I+G (parameter for gamma distribution = 1.0913). Maximum likelihood (ML) tree reconstruction with the best model and 100 bootstrap pseudoreplicates was run in MEGA 5 [106], [107]. Plants species and accession numbers are listed in Table S1.

### Mutagenesis of DgBRC1-1

Mutagenesis through PCR-driven overlap extension of *DgBRC1-1* was performed as previously described [108]. The nucleotides encoding the extra 17 amino acids in C-terminal of *DgBRC1-1* were frameshifted, and then cloned into pEZS-NL for further use in subcellular localization observations.

### Subcellular localization

For construction of the 35S::DgBRC1-GFP reporter plasmids, the ORFs of *DgBRC1-1* and *DgBRC1-2* were each cloned into binary vector pEZS-NL. Transformation into Onion (*Allium cepa*) was performed as described previously [109]. Onion peels were unfolded in water, and then viewed with an Eclipse C1si confocal microscope (Nikon); images were aquired using the EZ-C1 FreeViewer software (Nikon). GFP was visualized by using an excitation wavelength of 488 nm and a band pass 510 to 525 nm emission filter.

### Generation of transgenic plants

For complementation experiments, the ORFs of *DgBRC1-1* and *DgBRC1-2* were each cloned into binary vector pBI121, fusing them with the 35S promoter. The resulting constructs were transformed into Arabidopsis thaliana mutant *brc1* plants via *Agrobacteriaum tumefaciens* strain LBA4404 using the floral dip method [110], [111]. Independent transformants were screened on MS medium containing 50 mg l^−1^ kanamycin. Independent homozygous T3 lines with single insertion sites were used for the branching phenotype analysis.

### Split-plate and two-bud section system

The split-plate system was modified according to [46]. We used 30 ml MS medium for each 9 cm petri dish; after solidification, a 10 mm wide strip of the medium was removed from the centre of the plate. The volume of the media block remaining on each side was about 12.5 ml; 25 µl of 2.5 mM stock solutions containing various compounds were injected into one or both sides of the media blocks with a micro-pipette, and the final PGR concentration was 5 µM. BAP (Sigma B3408), NAA (Sigma N0640) and NPA (Dikma 46154) were dissolved in 70% ethanol, and GR24 (LeadGen Labs, Orangen, CT, USA) was dissolved in acetone. Stocks were stored at −20°C, and fresh stocks were made every month. Chrysanthemum seedlings were grown to10 cm high in sterile conditions, and then they were used for effects of PGRs on outgrowth of lateral branches and determination of *DgBRC1* transcripts in nodes. After PGRs had diffused evenly throughout the media, two-bud sections containing buds 3 and 4 were cut from the chrysanthemum seedlings, and then inserted into media. The total length of the two-bud sections was about 1.7 cm, and the distance between node 3 and node 4 was about 1.0 cm. It was ensured that at least 4 mm of the cut tips on each side were embedded into the media. The petri dishes were then held vertically in the room where intact plants were cultured. For each treatment, 8 plants were measured for growth of lateral branches every 24 h for 10 days by aligning a ruler behind the plates. Nodes 3 and 4 were harvested separately 4 h or 6 h after treatment for analysis of *DgBRC1* or *DgIPT3* transcripts. For each treatment, 10 plants were analyzed; all experiments were repeated for 3 biological replicates.

### Analysis of gene expression

Plant tissues were harvested and total RNA was isolated using TRIzol Reagent (Invitrogen). Traces of DNA were digested using 5 units of DNaseI (Takara, D2270A) for 30 min, followed by extraction by Phenol/Trichloromethane. cDNA was synthesized with the Quantscript RT Kit (Tiangen, KR103-04) using Nonadeoxyribonucleotide Mixture (Takara, D3802). The cDNA was diluted 1∶4 with water, and quantitative PCR was performed in 20 µl reactions with SYBR® Premix Ex Taq™ II (Takara, DRR081A) and the Applied Biosystems 7500 real-time PCR system according to the manufacturer's instructions. Ct values were obtained with the 7500 Systems SDS software 2.0.1 (Applied Biosystems). A standard curve for each target gene was generated using a dilution series of known concentrations of plasmid vectors containing target genes and measured by the same quantitative PCR process. Target Ct values were converted to absolute transcript numbers per reaction, and normalized to 18S rRNA transcript levels. Three biological replicates were measured for each treatment, and each replicate represented the RNA extracted from a pool of 10 buds. Primer sets are given in Table S3. The primers for total *DgBRC1* transcripts analysis were picked from the same part of *DgBRC1-1* and *DgBRC1-2*, while the sense or antisense primers for *DgBRC1-1* were selected exactly in the position where intron I was spliced.

### Statistics

ANOVA followed by a Duncan's test (for more than two comparisons) or a t test (two comparisons) was used (SPSS 18.0) where differences between means were assessed and significance was determined at α = 0.05.

## Supporting Information

Figure S1 **The length of all lateral branches in chrysanthemum plants of 45 cm, 65 cm, and 85 cm height.** Position of branches was recorded acropetally. Data were means ± SE; n = 20.
(TIF)

Figure S2 **Elongation of branches after decapitation (A) and flowering transition (B).** (A) Overall height of the top 10 branches 15 days after decapitation during vegetative period. (B) Length of flowering branches 15 days after flowering transition. Position of branches indicates branches which were numbered and recorded basipetally. Statistical comparisons were made within the length of intact and decapitated branches; asterisks indicate significant differences between branches at α = 0.05. Data are means ± SE. n = 11 to 16.
(TIF)

Figure S3 **Conserved TCP, R and ECE domains in DgBRC1 and other CYC proteins.** Alignment of the sequence encoding TCP domain to the R domain is presented. Identical amino acids are in black and amino acids with similar properties are in grey. The regions typical of TCP domain Basic-Helix I-Loop-Helix II are indicated. Dots denote the putative variable length of Helix II. The R domain and ECE domains present in the CYC1 subclade are indicated by red boxes. Sequences were aligned with ClustalW2 [91] and represented with Genedoc [91].
(TIF)

Figure S4 **Split plate system, and nodal sections including bud 3 and bud 4 for PGR treatment.**
(TIF)

Table S1 **Species and their corresponding accession numbers used to construct the phylogenetic tree.**
(DOC)

Table S2 **Rosette and cauline branch numbers in WT,** ***brc1-1*** **, and transgenic lines. R-bran, rosette branch; C-bran, cauline branch.**
(DOC)

Table S3 **Oligos cited in Materials and Methods.**
(DOC)

## References

[1] Laufs P, Grandjean O, Jonak C, Kiêu K, Traas J (1998) Cellular Parameters of the Shoot Apical Meristem in Arabidopsis. The Plant Cell Online 10: 1375–1390. 10.1105/tpc.10.8.1375 PMC144064 9707536

[2] McSteen P, Leyser O (2005) Shoot Branching. Annual Review of Plant Biology 56: 353–374. 10.1146/annurev.arplant.56.032604.144122 15862100

[3] Garrison R (1955) Studies in the Development of Axillary Buds. American Journal of Botany 42: 257–266.

[4] Mary Snow RS (1942) The Determination of Axillary Buds. New Phytologist 41: 13–22.

[5] Aguilar-Martinez JA, Poza-Carrion C, Cubas P (2007) Arabidopsis BRANCHED1 Acts as an Integrator of Branching Signals within Axillary Buds. The Plant Cell Online 19: 458–472. 10.1105/tpc.106.048934 PMC1867329 17307924

[6] Greb T, Clarenz O, Schäfer E, Müller D, Herrero R, et al (2003) Molecular analysis of the LATERAL SUPPRESSOR gene in Arabidopsis reveals a conserved control mechanism for axillary meristem formation. Genes & Development 17: 1175–1187. 12730136 10.1101/gad.260703 PMC196050

[7] Müller D (2006) Blind Homologous R2R3 Myb Genes Control the Pattern of Lateral Meristem Initiation in Arabidopsis. The Plant Cell Online 18: 586–597. 10.1105/tpc.105.038745 PMC1383635 16461581

[8] Yang F, Wang Q, Schmitz G, Müller D, Theres K (2012) The bHLH protein ROX acts in concert with RAX1 and LAS to modulate axillary meristem formation in Arabidopsis. The Plant Journal 71: 61–70. 22372440 10.1111/j.1365-313X.2012.04970.x

[9] Tantikanjana T (2001) Control of axillary bud initiation and shoot architecture in Arabidopsis through the SUPERSHOOT gene. Genes & Development 15: 1577–1588. 11410537 10.1101/gad.887301 PMC312715

[10] Thimann KV, Skoog F (1933) Studies on the Growth Hormone of Plants. III. The Inhibiting Action of the Growth Substance on Bud Development. Proceedings of the National Academy of Sciences of the United States of America 19: 714–716. 16577553 10.1073/pnas.19.7.714 PMC1086139

[11] Goldsmith MHM (1977) The Polar Transport of Auxin. Annual Review of Plant Physiology 28: 439–478.

[12] Booker J, Chatfield S, Leyser O (2003) Auxin Acts in Xylem-Associated or Medullary Cells to Mediate Apical Dominance. The Plant Cell Online 15: 495–507. 10.1105/tpc.007542 PMC141216 12566587

[13] Hillman JR, Math VB, Medlow GC (1977) Apical dominance and the levels of indole acetic acid in Phaseolus lateral buds. Planta 134: 191–193. 24419699 10.1007/BF00384970

[14] Eklöf S, Åstot C, Blackwell J, Moritz T, Olsson O, et al (1997) Auxin-Cytokinin Interactions in Wild-Type and Transgenic Tobacco. Plant and Cell Physiology 38: 225–235.

[15] Nordström A, Tarkowski P, Tarkowska D, Norbaek R, Åstot C, et al (2004) Auxin regulation of cytokinin biosynthesis in Arabidopsis thaliana: A factor of potential importance for auxin–cytokinin-regulated development. Proceedings of the National Academy of Sciences of the United States of America 101: 8039–8044. 15146070 10.1073/pnas.0402504101 PMC419553

[16] Brewer PB, Dun EA, Ferguson BJ, Rameau C, Beveridge CA (2009) Strigolactone Acts Downstream of Auxin to Regulate Bud Outgrowth in Pea and Arabidopsis. Plant Physiology 150: 482–493. 19321710 10.1104/pp.108.134783 PMC2675716

[17] Li C-J, Bangerth F (1999) Autoinhibition of indoleacetic acid transport in the shoots of two-branched pea (Pisum sativum) plants and its relationship to correlative dominance. Physiologia Plantarum 106: 415–420.

[18] Bennett T, Sieberer T, Willett B, Booker J, Luschnig C, et al (2006) The Arabidopsis MAX Pathway Controls Shoot Branching by Regulating Auxin Transport. Current Biology 16: 553–563. 16546078 10.1016/j.cub.2006.01.058

[19] Prusinkiewicz P, Crawford S, Smith RS, Ljung K, Bennett T, et al (2009) Control of bud activation by an auxin transport switch. Proceedings of the National Academy of Sciences 106: 17431–17436. 10.1073/pnas.0906696106 PMC2751654 19805140

[20] Renton M, Hanan J, Ferguson BJ, Beveridge CA (2012) Models of long-distance transport: how is carrier-dependent auxin transport regulated in the stem? New Phytologist 194: 704–715. 22443265 10.1111/j.1469-8137.2012.04093.x

[21] Dun EA, Hanan J, Beveridge CA (2009) Computational Modeling and Molecular Physiology Experiments Reveal New Insights into Shoot Branching in Pea. The Plant Cell Online 21: 3459–3472. 10.1105/tpc.109.069013 PMC2798318 19948786

[22] Balla J, Kalousek P, Reinöhl V, Friml J, Procházka S (2011) Competitive canalization of PIN-dependent auxin flow from axillary buds controls pea bud outgrowth. The Plant Journal 65: 571–577. 21219506 10.1111/j.1365-313X.2010.04443.x

[23] Crawford S, Shinohara N, Sieberer T, Williamson L, George G, et al (2010) Strigolactones enhance competition between shoot branches by dampening auxin transport. Development 137: 2905–2913. 20667910 10.1242/dev.051987

[24] Dun EA, de Saint Germain A, Rameau C, Beveridge CA (2013) Dynamics of Strigolactone Function and Shoot Branching Responses in Pisum sativum. Molecular Plant 6: 128–140. 23220942 10.1093/mp/sss131

[25] Gomez-Roldan V, Fermas S, Brewer PB, Puech-Pages V, Dun EA, et al (2008) Strigolactone inhibition of shoot branching. Nature 455: 189–194. 18690209 10.1038/nature07271

[26] Umehara M, Hanada A, Yoshida S, Akiyama K, Arite T, et al (2008) Inhibition of shoot branching by new terpenoid plant hormones. Nature 455: 195–200. 18690207 10.1038/nature07272

[27] Stirnberg P, Furner IJ, Ottoline Leyser HM (2007) MAX2 participates in an SCF complex which acts locally at the node to suppress shoot branching. The Plant Journal 50: 80–94. 17346265 10.1111/j.1365-313X.2007.03032.x

[28] Stirnberg P, van de Sande K, Leyser HMO (2002) MAX1 and MAX2 control shoot lateral branching in Arabidopsis. Development 129: 1131–1141. 11874909 10.1242/dev.129.5.1131

[29] Booker J, Auldridge M, Wills S, McCarty D, Klee H, et al (2004) MAX3/CCD7 Is a Carotenoid Cleavage Dioxygenase Required for the Synthesis of a Novel Plant Signaling Molecule. Current Biology 14: 1232–1238. 15268852 10.1016/j.cub.2004.06.061

[30] Booker J, Sieberer T, Wright W, Williamson L, Willett B, et al (2005) MAX1 Encodes a Cytochrome P450 Family Member that Acts Downstream of MAX3/4 to Produce a Carotenoid-Derived Branch-Inhibiting Hormone. Developmental Cell 8: 443–449. 15737939 10.1016/j.devcel.2005.01.009

[31] Simons JL, Napoli CA, Janssen BJ, Plummer KM, Snowden KC (2007) Analysis of the DECREASED APICAL DOMINANCE Genes of Petunia in the Control of Axillary Branching. Plant Physiology 143: 697–706. 17158589 10.1104/pp.106.087957 PMC1803742

[32] Snowden KC, Simkin AJ, Janssen BJ, Templeton KR, Loucas HM, et al (2005) The Decreased apical dominance1/Petunia hybrida CAROTENOID CLEAVAGE DIOXYGENASE8 Gene Affects Branch Production and Plays a Role in Leaf Senescence, Root Growth, and Flower Development. The Plant Cell Online 17: 746–759. 10.1105/tpc.104.027714 PMC1069696 15705953

[33] Drummond RSM, Martínez-Sánchez NM, Janssen BJ, Templeton KR, Simons JL, et al (2009) Petunia hybrida CAROTENOID CLEAVAGE DIOXYGENASE7 Is Involved in the Production of Negative and Positive Branching Signals in Petunia. Plant Physiology 151: 1867–1877. 19846541 10.1104/pp.109.146720 PMC2785980

[34] Sorefan K, Booker J, Haurogné K, Goussot M, Bainbridge K, et al (2003) MAX4 and RMS1 are orthologous dioxygenase-like genes that regulate shoot branching in Arabidopsis and pea. Genes & Development 17: 1469–1474. 12815068 10.1101/gad.256603 PMC196077

[35] Beveridge CA, Ross JJ, Murfet IC (1996) Branching in Pea (Action of Genes Rms3 and Rms4). Plant Physiology 110: 859–865. 12226224 10.1104/pp.110.3.859 PMC157785

[36] Beveridge CA, Symons GM, Murfet IC, Ross JJ, Rameau C (1997) The rms1 Mutant of Pea Has Elevated Indole-3-Acetic Acid Levels and Reduced Root-Sap Zeatin Riboside Content but Increased Branching Controlled by Graft-Transmissible Signal(s). Plant Physiology 115: 1251–1258.

[37] Foo E, Turnbull CGN, Beveridge CA (2001) Long-Distance Signaling and the Control of Branching in the rms1 Mutant of Pea. Plant Physiology 126: 203–209. 11351083 10.1104/pp.126.1.203 PMC102294

[38] Arite T, Iwata H, Ohshima K, Maekawa M, Nakajima M, et al (2007) DWARF10, an RMS1/MAX4/DAD1 ortholog, controls lateral bud outgrowth in rice. The Plant Journal 51: 1019–1029. 17655651 10.1111/j.1365-313X.2007.03210.x

[39] Arite T, Umehara M, Ishikawa S, Hanada A, Maekawa M, et al (2009) d14, a Strigolactone-Insensitive Mutant of Rice, Shows an Accelerated Outgrowth of Tillers. Plant and Cell Physiology 50: 1416–1424. 19542179 10.1093/pcp/pcp091

[40] Liang J, Zhao L, Challis R, Leyser O (2010) Strigolactone regulation of shoot branching in chrysanthemum (Dendranthema grandiflorum). Journal of Experimental Botany 61: 3069–3078. 20478970 10.1093/jxb/erq133 PMC2892150

[41] Ferguson BJ, Beveridge CA (2009) Roles for Auxin, Cytokinin, and Strigolactone in Regulating Shoot Branching. Plant Physiology 149: 1929–1944. 19218361 10.1104/pp.109.135475 PMC2663762

[42] Rasmussen A, Mason M, De Cuyper C, Brewer PB, Herold S, et al (2012) Strigolactones suppress adventitious rooting in Arabidopsis and pea. Plant Physiology 10.1104/pp.111.187104 PMC3320200 22323776

[43] Kapulnik Y, Resnick N, Mayzlish-Gati E, Kaplan Y, Wininger S, et al (2011) Strigolactones interact with ethylene and auxin in regulating root-hair elongation in Arabidopsis. Journal of Experimental Botany 62: 2915–2924. 21307387 10.1093/jxb/erq464

[44] Agusti J, Herold S, Schwarz M, Sanchez P, Ljung K, et al (2011) Strigolactone signaling is required for auxin-dependent stimulation of secondary growth in plants. Proceedings of the National Academy of Sciences 108: 20242–20247. 10.1073/pnas.1111902108 PMC3250165 22123958

[45] Sachs T, Thimann KV (1967) The Role of Auxins and Cytokinins in the Release of Buds From Dominance. American Journal of Botany 54: 136–144.

[46] Chatfield SP, Stirnberg P, Forde BG, Leyser O (2000) The hormonal regulation of axillary bud growth in Arabidopsis. The Plant Journal 24: 159–169. 11069691 10.1046/j.1365-313x.2000.00862.x

[47] Cline M (1991) Apical dominance. The Botanical Review 57: 318–358.

[48] Tanaka M, Takei K, Kojima M, Sakakibara H, Mori H (2006) Auxin controls local cytokinin biosynthesis in the nodal stem in apical dominance. The Plant Journal 45: 1028–1036. 16507092 10.1111/j.1365-313X.2006.02656.x

[49] Jones B, Gunnerås SA, Petersson SV, Tarkowski P, Graham N, et al (2010) Cytokinin Regulation of Auxin Synthesis in Arabidopsis Involves a Homeostatic Feedback Loop Regulated via Auxin and Cytokinin Signal Transduction. The Plant Cell Online 10.1105/tpc.110.074856 PMC2965550 20823193

[50] Dun EA, Brewer PB, Beveridge CA (2009) Strigolactones: discovery of the elusive shoot branching hormone. Trends in Plant Science 14: 364–372. 19540149 10.1016/j.tplants.2009.04.003

[51] Domagalska MA, Leyser O (2011) Signal integration in the control of shoot branching. Nat Rev Mol Cell Biol 12: 211–221. 21427763 10.1038/nrm3088

[52] Ongaro V, Leyser O (2007) Hormonal control of shoot branching. Journal of Experimental Botany 59: 67–74. 17728300 10.1093/jxb/erm134

[53] Doebley J, Stec A, Hubbard L (1997) The evolution of apical dominance in maize. Nature 386: 485–488. 9087405 10.1038/386485a0

[54] Takeda T, Suwa Y, Suzuki M, Kitano H, Ueguchi-Tanaka M, et al (2003) The OsTB1 gene negatively regulates lateral branching in rice. The Plant Journal 33: 513–520. 12581309 10.1046/j.1365-313x.2003.01648.x

[55] Kebrom TH, Burson BL, Finlayson SA (2006) Phytochrome B Represses Teosinte Branched1 Expression and Induces Sorghum Axillary Bud Outgrowth in Response to Light Signals. Plant Physiology 140: 1109–1117. 16443694 10.1104/pp.105.074856 PMC1400571

[56] Martín-Trillo M, Grandío EG, Serra F, Marcel F, Rodríguez-Buey ML, et al (2011) Role of tomato BRANCHED1-like genes in the control of shoot branching. The Plant Journal 67: 701–714. 21554455 10.1111/j.1365-313X.2011.04629.x

[57] Finlayson SA (2007) Arabidopsis TEOSINTE BRANCHED1-LIKE 1 Regulates Axillary Bud Outgrowth and is Homologous to Monocot TEOSINTE BRANCHED1. Plant and Cell Physiology 48: 667–677. 17452340 10.1093/pcp/pcm044

[58] Doebley J, Stec A, Gustus C (1995) teosinte branched1 and the Origin of Maize: Evidence for Epistasis and the Evolution of Dominance. Genetics 141: 333–346. 8536981 10.1093/genetics/141.1.333 PMC1206731

[59] Minakuchi K, Kameoka H, Yasuno N, Umehara M, Luo L, et al (2010) FINE CULM1 (FC1) Works Downstream of Strigolactones to Inhibit the Outgrowth of Axillary Buds in Rice. Plant and Cell Physiology 51: 1127–1135. 20547591 10.1093/pcp/pcq083 PMC2900823

[60] Braun N, de Saint Germain A, Pillot J-P, Boutet-Mercey S, Dalmais M, et al (2012) The Pea TCP Transcription Factor PsBRC1 Acts Downstream of Strigolactones to Control Shoot Branching. Plant Physiology 158: 225–238. 22045922 10.1104/pp.111.182725 PMC3252107

[61] Whipple CJ, Kebrom TH, Weber AL, Yang F, Hall D, et al (2011) grassy tillers1 promotes apical dominance in maize and responds to shade signals in the grasses. Proceedings of the National Academy of Sciences 10.1073/pnas.1102819108 PMC3158142 21808030

[62] Yu J, Dong L, Xi L, Zhao R, Ma N, et al (2012) Isolation and Characterization of Cytokinin Synthase Gene DgIPT3 in Chrysanthemum ‘Jinba’. Acta Horticulturae Sinica 39: 712–728.

[63] Jiang B, Miao H, Chen S, Zhang S, Chen F, et al (2009) The Lateral Suppressor-Like Gene, DgLsL, Alternated the Axillary Branching in Transgenic Chrysanthemum (Chrysanthemum×morifolium) by Modulating IAA and GA Content. Plant Molecular Biology Reporter 28: 144–151.

[64] Han B, Suh E, Lee S, Shin H, Lim Y (2007) Selection of non-branching lines induced by introducing Ls -like cDNA into Chrysanthemum (Dendranthema×grandiflorum (Ramat.) Kitamura) “Shuho-no-chikara”. Scientia Horticulturae 115: 70–75.

[65] Cline M (1997) Concepts and terminology of apical dominance. American Journal of Botany 84: 1064. 21708661

[66] Cubas P, Lauter N, Doebley J, Coen E (1999) The TCP domain: a motif found in proteins regulating plant growth and development. The Plant Journal 18: 215–222. 10363373 10.1046/j.1365-313x.1999.00444.x

[67] Wang C, Chen J, Jongsma MA (2004) Molecular Evolution and Phylogeny of Florist's Chrysanthemum and Related Species. Journal of Beijing Forestry University 26: 91–96.

[68] Kosugi S, Ohashi Y (1997) PCF1 and PCF2 Specifically Bind to cis Elements in the Rice Proliferating Cell Nuclear Antigen Gene. The Plant Cell Online 9: 1607–1619. 10.1105/tpc.9.9.1607 PMC157037 9338963

[69] Suzuki T, Sakurai K, Ueguchi C, Mizuno T (2001) Two types of putative nuclear factors that physically interact with histidine-containing phosphotransfer (Hpt) domains, signaling mediators in His-to-Asp phosphorelay, in Arabidopsis thaliana. Plant Cell Physiol 42: 37–45. 11158442 10.1093/pcp/pce011

[70] Qin L, Guo X, Feng X, Weng L, Yan J, et al (2004) Cloning of LjCYC1 gene and nuclear localization of LjCYC1 protein in Lotus japonicus. Journal of Plant Physiology and Molecular Biology 30: 523–532. 15627706

[71] Smith H (1995) Physiological and Ecological Function within the Phytochrome Family. Annual Review of Plant Physiology and Plant Molecular Biology 46: 289–315.

[72] Franklin KA, Whitelam GC (2005) Phytochromes and Shade-avoidance Responses in Plants. Annals of Botany 96: 169–175. 15894550 10.1093/aob/mci165 PMC4246865

[73] Casal JJ, Sanchez RA, Deregibus VA (1986) The effect of plant density on tillering: The involvement of R/FR ratio and the proportion of radiation intercepted per plant. Environmental and Experimental Botany 26: 365–371.

[74] Finlayson SA, Krishnareddy SR, Kebrom TH, Casal JJ (2010) Phytochrome Regulation of Branching in Arabidopsis. Plant Physiology 152: 1914–1927. 20154098 10.1104/pp.109.148833 PMC2850038

[75] Howarth DG, Donoghue MJ (2006) Phylogenetic analysis of the “ECE” (CYC/TB1) clade reveals duplications predating the core eudicots. Proceedings of the National Academy of Sciences 103: 9101–9106. 10.1073/pnas.0602827103 PMC1482573 16754863

[76] Martín-Trillo M, Cubas P (2010) TCP genes: a family snapshot ten years later. Trends in Plant Science 15: 31–39. 19963426 10.1016/j.tplants.2009.11.003

[77] Luo D, Carpenter R, Copsey L, Vincent C, Clark J, et al (1999) Control of organ asymmetry in flowers of Antirrhinum. Cell 99: 367–376. 10571179 10.1016/s0092-8674(00)81523-8

[78] Nath U, Crawford BCW, Carpenter R, Coen E (2003) Genetic Control of Surface Curvature. Science 299: 1404–1407. 12610308 10.1126/science.1079354

[79] Palatnik JF, Allen E, Wu X, Schommer C, Schwab R, et al (2003) Control of leaf morphogenesis by microRNAs. Nature 425: 257–263. 12931144 10.1038/nature01958

[80] Broholm SK, Tähtiharju S, Laitinen RAE, Albert VA, Teeri TH, et al (2008) A TCP domain transcription factor controls flower type specification along the radial axis of the Gerbera (Asteraceae) inflorescence. Proceedings of the National Academy of Sciences 105: 9117–9122. 10.1073/pnas.0801359105 PMC2449374 18574149

[81] Da L, Carpenter R, Copsey L, Vincent C, Clark J, et al (1999) Control of Organ Asymmetry in Flowers of Antirrhinum. Cell 99: 367–376. 10571179 10.1016/s0092-8674(00)81523-8

[82] Luo D, Carpenter R, Vincent C, Copsey L, Coen E (1996) Origin of floral asymmetry in Antirrhinum. Nature 383: 794–799. 8893002 10.1038/383794a0

[83] Chapman MA, Leebens-Mack JH, Burke JM (2008) Positive Selection and Expression Divergence Following Gene Duplication in the Sunflower CYCLOIDEA Gene Family. Molecular Biology and Evolution 25: 1260–1273. 18390478 10.1093/molbev/msn001

[84] Amor S, Remy S, Dambrine G, Le Vern Y, Rasschaert D, et al (2010) Alternative splicing and nonsense-mediated decay regulate telomerase reverse transcriptase (TERT) expression during virus-induced lymphomagenesis in vivo. BMC Cancer 10: 571. 20964812 10.1186/1471-2407-10-571 PMC2976754

[85] Kertész S, Kerényi Z, Mérai Z, Bartos I, Pálfy T, et al (2006) Both introns and long 3′-UTRs operate as cis-acting elements to trigger nonsense-mediated decay in plants. Nucleic Acids Research 34: 6147–6157. 17088291 10.1093/nar/gkl737 PMC1693880

[86] Koroleva OA, Tomlinson ML, Leader D, Shaw P, Doonan JH (2005) High-throughput protein localization in Arabidopsis using Agrobacterium-mediated transient expression of GFP-ORF fusions. The Plant Journal 41: 162–174. 15610358 10.1111/j.1365-313X.2004.02281.x

[87] Kudo T, Kiba T, Sakakibara H (2010) Metabolism and Long-distance Translocation of Cytokinins. Journal of Integrative Plant Biology 52: 53–60. 20074140 10.1111/j.1744-7909.2010.00898.x

[88] Ali A, Fletcher RA (1971) Hormonal interaction in controlling apical dominance in soybeans. Canadian Journal of Botany 49: 1727–1731.

[89] Davies CR, Seth AK, Wareing PF (1966) Auxin and Kinetin Interaction in Apical Dominance. Science 151: 468–469. 17798523 10.1126/science.151.3709.468

[90] Wickson M, Thimann KV (1958) The Antagonism of Auxin and Kinetin in Apical Dominance. Physiologia Plantarum 11: 62–74.

[91] Shein T, Jackson DI (1972) Interaction between Hormones, Light, and Nutrition on Extension of Lateral Buds in Phaseolus vulgaris L. Annals of Botany 36: 791–800.

[92] Kalousek P, Buchtová D, Balla J, Reinöhl V, Procházka S (2010) Cytokinins and polar transport of auxin in axillary pea buds. ACTA UNIVERSITATIS AGRICULTURAE ET SILVICULTURAE MENDELIANAE BRUNENSIS 58: 79–88.

[93] Müller D, Leyser O (2011) Auxin, cytokinin and the control of shoot branching. Annals of Botany 107: 1203–1212. 21504914 10.1093/aob/mcr069 PMC3091808

[94] Kebrom TH, Brutnell TP, Hays DB, Finlayson SA (2010) Vegetative axillary bud dormancy induced by shade and defoliation signals in the grasses. Plant Signaling & Behavior 5: 317–319. 20200487 10.4161/psb.5.3.11186 PMC2881289

[95] Bangerth F (1994) Response of cytokinin concentration in the xylem exudate of bean (Phaseolus vulgaris L.) plants to decapitation and auxin treatment, and relationship to apical dominance. Planta 194: 439–442.

[96] Turnbull CGN, Raymond MAA, Dodd IC, Morris SE (1997) Rapid increases in cytokinin concentration in lateral buds of chickpea (Cicer arietinum L.) during release of apical dominance. Planta 202: 271–276.

[97] Morris DA, Winfield PJ (1972) Kinetin Transport to Axillary Buds of Dwarf Pea (Pisum sativum L.). Journal of Experimental Botany 23: 346–354.

[98] Ando S, Asano T, Tsushima S, Kamachi S, Hagio T, et al (2005) Changes in gene expression of putative isopentenyltransferase during clubroot development in Chinese cabbage (Brassica rapa L.). Physiological and Molecular Plant Pathology 67: 59–67.

[99] Brewer PB, Koltai H, Beveridge CA (2013) Diverse Roles of Strigolactones in Plant Development. Molecular Plant 6: 18–28. 23155045 10.1093/mp/sss130

[100] Bishopp A, Benková E, Helariutta Y (2011) Sending mixed messages: auxin-cytokinin crosstalk in roots. Current Opinion in Plant Biology 14: 10–16. 20926335 10.1016/j.pbi.2010.08.014

[101] Hayward A, Stirnberg P, Beveridge C, Leyser O (2009) Interactions between Auxin and Strigolactone in Shoot Branching Control. Plant Physiology 151: 400–412. 19641034 10.1104/pp.109.137646 PMC2735998

[102] Murashige T, Skoog F (1962) A Revised Medium for Rapid Growth and Bio Assays with Tobacco Tissue Cultures. Physiologia Plantarum 15: 473–497.

[103] Edgar RC (2004) MUSCLE: multiple sequence alignment with high accuracy and high throughput. Nucleic Acids Research 32: 1792–1797. 15034147 10.1093/nar/gkh340 PMC390337

[104] Nicholas KB, Nicholas HB, Deerfield DW (1997) GeneDoc: analysis and visualization of genetic variation. EMBNEW NEWS 4: 14.

[105] Posada D (2008) jModelTest: Phylogenetic Model Averaging. Molecular Biology and Evolution 25: 1253–1256. 18397919 10.1093/molbev/msn083

[106] Guindon S, Gascuel O (2003) A Simple, Fast, and Accurate Algorithm to Estimate Large Phylogenies by Maximum Likelihood. Systematic Biology 52: 696–704. 14530136 10.1080/10635150390235520

[107] Tamura K, Peterson D, Peterson N, Stecher G, Nei M, et al (2011) MEGA5: Molecular Evolutionary Genetics Analysis using Maximum Likelihood, Evolutionary Distance, and Maximum Parsimony Methods. Molecular Biology and Evolution 28: 2731–2739. 21546353 10.1093/molbev/msr121 PMC3203626

[108] Ho SN, Hunt HD, Horton RM, Pullen JK, Pease LR (1989) Site-directed mutagenesis by overlap extension using the polymerase chain reaction. Gene 77: 51–59. 2744487 10.1016/0378-1119(89)90358-2

[109] Varagona MJ, Schmidt RJ, Raikhel NV (1992) Nuclear Localization Signal(s) Required for Nuclear Targeting of the Maize Regulatory Protein Opaque-2. The Plant Cell Online 4: 1213–1227. 10.1105/tpc.4.10.1213 PMC160209 1332794

[110] Clough SJ, Bent AF (1998) Floral dip: a simplified method forAgrobacterium-mediated transformation of Arabidopsis thaliana. The Plant Journal 16: 735–743. 10069079 10.1046/j.1365-313x.1998.00343.x

[111] Zhang X, Henriques R, Lin S-S, Niu Q-W, Chua N-H (2006) Agrobacterium-mediated transformation of Arabidopsis thaliana using the floral dip method. Nat Protocols 1: 641–646. 17406292 10.1038/nprot.2006.97

