# Supplementary figures and images for: Roles of DgBRC1 in Regulation of Lateral Branching in Chrysanthemum (Dendranthema ×grandiflora cv. Jinba)
Source: PLoS One. 2013 Apr 17;8(4):e61717. doi: 10.1371/journal.pone.0061717 (PMC3629106; doi:10.1371/journal.pone.0061717)

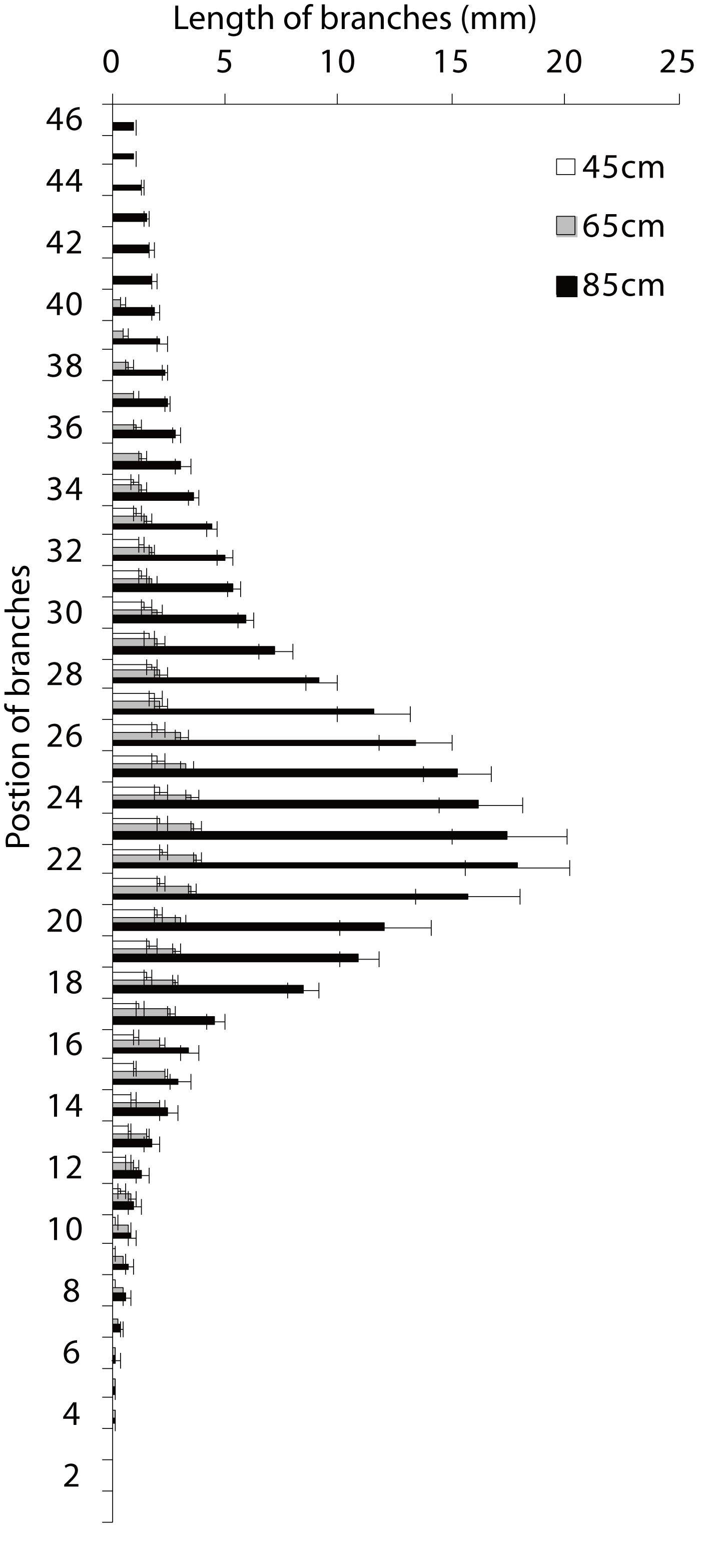

Supplement: Figure S1 — The length of all lateral branches in chrysanthemum plants of 45 cm, 65 cm, and 85 cm height. Position of branches was recorded acropetally. Data were means ± SE; n = 20. (TIF) [file pone.0061717.s001.tif]

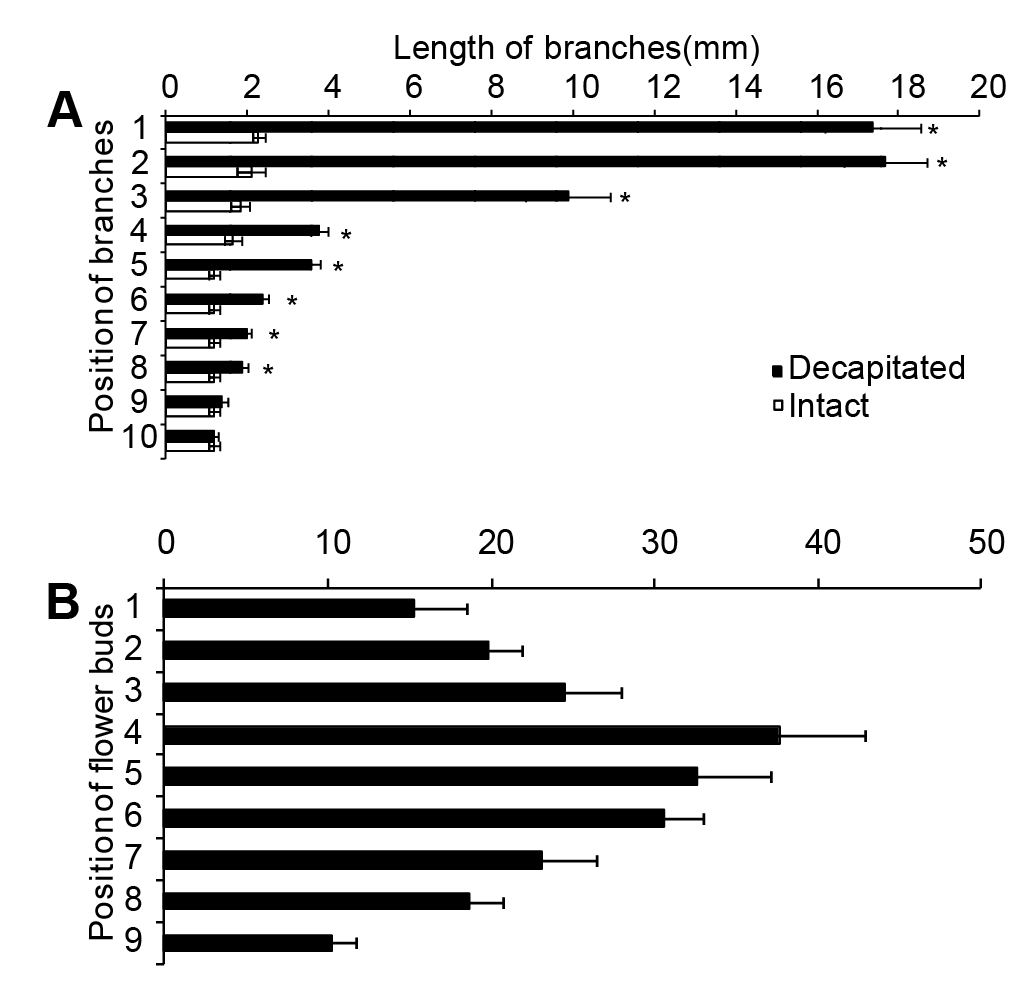

Supplement: Figure S2 — Elongation of branches after decapitation (A) and flowering transition (B). (A) Overall height of the top 10 branches 15 days after decapitation during vegetative period. (B) Length of flowering branches 15 days after flowering transition. Position of branches indicates branches which were numbered and recorded basipetally. Statistical comparisons were made within the length of intact and decapitated branches; asterisks indicate significant differences between branches at α = 0.05. Data are means ± SE. n = 11 to 16. (TIF) [file pone.0061717.s002.tif]

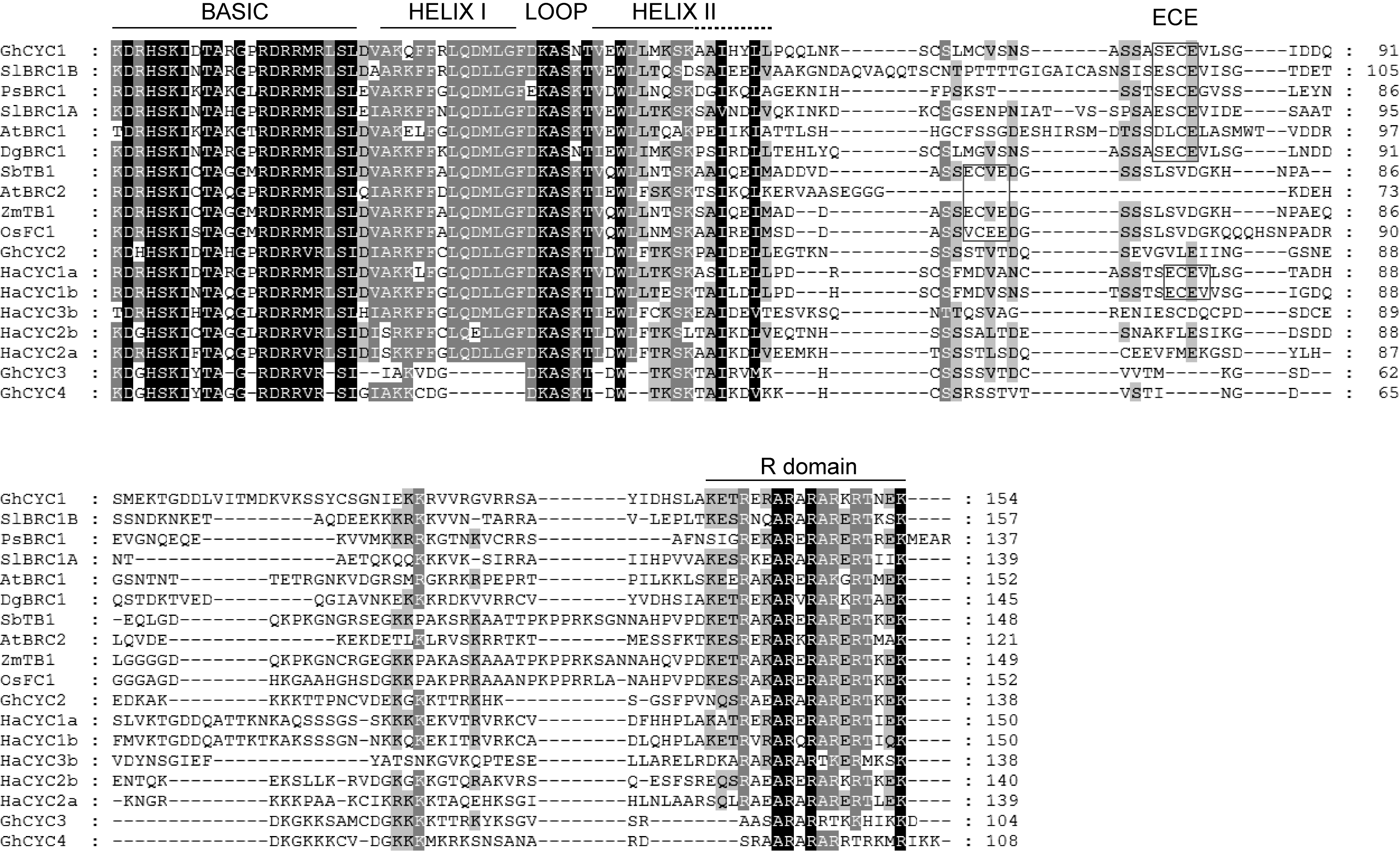

Supplement: Figure S3 — Conserved TCP, R and ECE domains in DgBRC1 and other CYC proteins. Alignment of the sequence encoding TCP domain to the R domain is presented. Identical amino acids are in black and amino acids with similar properties are in grey. The regions typical of TCP domain Basic-Helix I-Loop-Helix II are indicated. Dots denote the putative variable length of Helix II. The R domain and ECE domains present in the CYC1 subclade are indicated by red boxes. Sequences were aligned with ClustalW2 [91] and represented with Genedoc [91]. (TIF) [file pone.0061717.s003.tif]

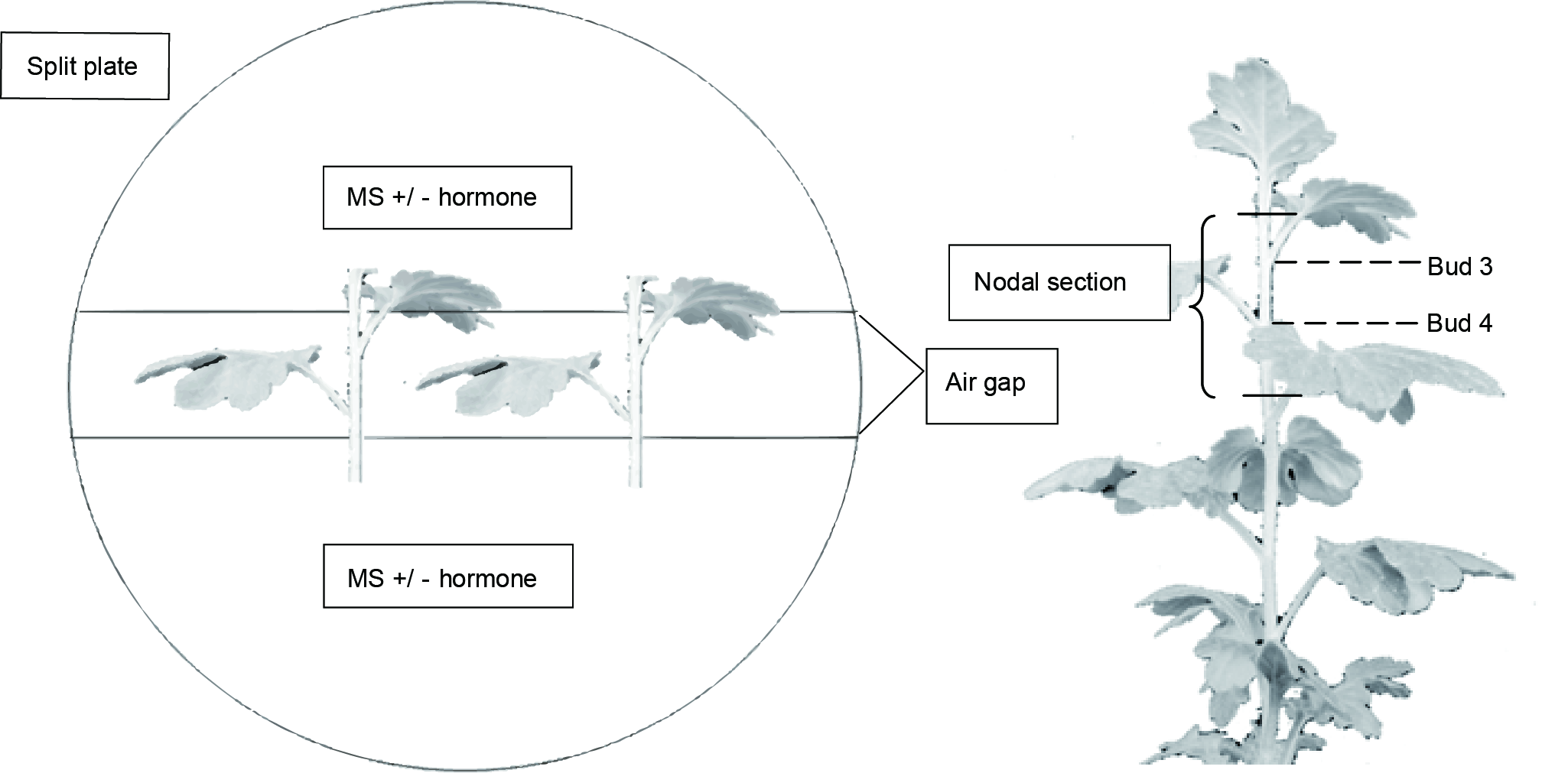

Supplement: Figure S4 — Split plate system, and nodal sections including bud 3 and bud 4 for PGR treatment. (TIF) [file pone.0061717.s004.tif]
