# Supplementary material for: Roles of DgBRC1 in Regulation of Lateral Branching in Chrysanthemum (Dendranthema ×grandiflora cv. Jinba)
Source: PLoS One. 2013 Apr 17;8(4):e61717. doi: 10.1371/journal.pone.0061717 (PMC3629106; doi:10.1371/journal.pone.0061717)
Supplement: Table S1 — Species and their corresponding accession numbers used to construct the phylogenetic tree. (DOC) [file pone.0061717.s005.doc]

**Table S1. Sequences used in the phylogenetic analysis of TCP genes**

| Species | Gene name | GenBank accession no. |
| --- | --- | --- |
| *Dendranthema ×grandiflora* cv ‘Jinba’ | *DgBRC1* | JX870411 |
| *Gerbera hybrida* | *GhCYC1* | EU429302 |
|  | *GhCYC2* | EU429303 |
|  | *GhCYC3* | EU429304 |
|  | *GhCYC4* | EU429305 |
|  | *GhCYC5* | JN190059 |
|  | *GhCYC6* | JN190060 |
|  | *GhCYC7* | JN190061 |
|  | *GhCYC9* | JN190063 |
|  | *GhCYC8* | JN190062 |
|  | *GhCYC10* | JN190064 |
| *Helianthus annuus* | *HaCYC1a* | EU088367 |
|  | *HaCYC1b* | EU088366 |
| *Arabidopsis thaliana* | *At BRC1* | AM408560 |
|  | *AtBRC2* | AM408561 |
|  | *AtTCP4* | NM_180258 |
| [*Solanum lycopersicum*](http://www.ncbi.nlm.nih.gov/nuccore/HM921067.1) | *SlBRC2b* | HM921067 |
|  | *SlBRC1b* | HM597230 |
|  | *SlBRC1a* | HM597229 |
|  | *SlBRC2a* | HM921066 |
| *Pisum sativum cultivar Terese* | *PsBRC1* | JF274232 |
| *Populus deltoides* | *PdTB1* | AF309094 |
| *Populus balsamifera* subsp. trichocarpa | *PbTB2* | AY012513 |
| *Zea mays teosinte* | *ZmTB1* | AF415152 |
| *Oryza sativa* | *OsFC1* | AF322143 |
