# Supplementary material for: Roles of DgBRC1 in Regulation of Lateral Branching in Chrysanthemum (Dendranthema ×grandiflora cv. Jinba)
Source: PLoS One. 2013 Apr 17;8(4):e61717. doi: 10.1371/journal.pone.0061717 (PMC3629106; doi:10.1371/journal.pone.0061717)
Supplement: Table S2 — Rosette and cauline branch numbers in WT, brc1-1 , and transgenic lines. R-bran, rosette branch; C-bran, cauline branch. (DOC) [file pone.0061717.s006.doc]

**Table S2. Rosette and cauline branch number in WT, *brc1-1*, and transgenic lines**

|  | *brc1-1* | 35S::DgBRC1variant 1 in *brc1-1* | | | | | 35S::DgBRC1variant 2 in *brc1-1* | | | | |
| --- | --- | --- | --- | --- | --- | --- | --- | --- | --- | --- | --- |
|  |  | Line 1 | Line 2 | Line 3 | Line 4 | Line 5 | Line 1 | Line 2 | Line 3 | Line 4 | Line 5 |
| R-bran | 7.63 | 4.55 | 4.67 | 4.45 | 4.86 | 4.75 | 5.44 | 5.32 | 5.70 | 5.52 | 5.16 |
| C-bran | 3.42 | 1.75 | 1.83 | 1.76 | 1.94 | 1.75 | 3.11 | 3.05 | 3.25 | 3.16 | 3.06 |
|  | WT | 35S::DgBRC1 variant 1 in WT | | | | | 35S::DgBRC1 variant 2 in WT | | | | |
|  |  | Line 1 | Line 2 | Line 3 | Line 4 | Line 5 | Line 1 | Line 2 | Line 3 | Line 4 | Line 5 |
| R-bran | 4.60 | 1.85 | 1.80 | 1.95 | 2.06 | 1.87 | 1.91 | 1.89 | 1.97 | 2.12 | 2.08 |
| C-bran | 2.61 | 1.97 | 1.84 | 1.88 | 1.97 | 1.97 | 2.09 | 1.96 | 2.10 | 2.08 | 2.03 |
