# Supplementary material for: Roles of DgBRC1 in Regulation of Lateral Branching in Chrysanthemum (Dendranthema ×grandiflora cv. Jinba)
Source: PLoS One. 2013 Apr 17;8(4):e61717. doi: 10.1371/journal.pone.0061717 (PMC3629106; doi:10.1371/journal.pone.0061717)
Supplement: Table S3 — Oligos cited in Materials and Methods. (DOC) [file pone.0061717.s007.doc]

**Table S3. Primers used.**

| Primer name | DNA sequence | Experiment |
| --- | --- | --- |
| BRC1-F1 | AGRAMRGACMGGCACAGCAAGAT | Fragment amplifying |
| BRC1-F2 | GGTATGAGAGATAGAAGAATGAGACTGTCTYTNGAYGTNGC | Fragment amplifying |
| BRC1-R1 | TCCATTGTTCTTTCTCTAGCTCTTTCNCKNGCYTT | Fragment amplifying |
| 3’RACE-AP | GGCCACGCGTCGACTAGTACT(17) | 3’RACE cDNA synthesis primer |
| BRC1-F3 | GCTTCAGCCTTATGGGTGTGTCA | 3’RACE |
| BRC1-F4 | TTCCGAATGTGAAGTTCTGTCTGG | 3’RACE |
| 5’RACE-AAP | GGCCACGCGTCGACTAGTACGGIIGGGIIGGGGIIG | 5’RACE |
| AUAP | GGCCACGCGTCGACTAGTACG | 5’RACE and 3’ RACE |
| BRC1-R2 | CTTGACACTGACCTATTTGC | 5’RACE cDNA synthesis primer |
| BRC1-R3 | TCCAAGACCCTAATCGGCTCATA | 5’RACE |
| BRC1-R4 | AACCACTCGATAGTATTGCTTGCC | 5’RACE |
| BRC1-F5 | CGGCATAGCTGGTCTTTGGACTAT | Full-length amplifying |
| BRC1-R5 | CACCCCTTGACACTGACCTATTTGC | Full-length amplifying |
| BRC1-F6 | CAGTCGACATGCATCCATCATTTTCCTCATT | pEZS-NL construction |
| BRC1-1-R1 | GATCCCGGGCTACCTCATGAGAAATTCCAAT | pEZS-NL construction |
| BRC1-2-R1 | GATCCCGGGCTTTGCTCATGCCTTCCCATGG | pEZS-NL construction |
| BRC1-F7 | GCTCTAGAGCATGCATCCATCATTTTCCTC | pBI121 construction |
| BRC1-1-R2 | CGGAGCTCCGTTATACCTCATGAGAAATTC | pBI121 construction |
| BRC1-2-R2 | GCGAGCTCGCCTATTTGCTCATGCCTTCCC | pBI121 construction |
| BRC1-2-Mu- R1 | GATGATCTCATGAGAAATTCCAATAT | Mutation of variant 2 |
| BRC1-2-Mu-F1 | ATATTGGAATTTCTCATGAGATCATC | Mutation of variant 2 |
| BRC1-2-Mu-R2 | GATCCCGGGCATTTGCTCTTGCCTTCCCATGG | Mutation of variant 2 |
| BRC1-F8 | CCCTTTTGGAGAGCATCAAG | QPCR |
| BRC1-R8 | AGACGTCGCGGATGAAGTAT | QPCR |
| BRC1-2-F3 | ATTTCTCATGAGCATCAGTTCAGT | QPCR |
| BRC1-2-R3 | GAGCAGTTTGAAGTTGCTTATCC | QPCR |
| IPT3-F1 | GGTATCCGCAGGTATTGTTGAA | QPCR |
| IPT3-R1 | ATTCTTCACGAAAATAAGCAT | QPCR |
| 18S-F1 | AAACGGCTACCACATCCAAG | QPCR |
| 18S-R1 | ACTCGAAAGAGCCCGGTATT | QPCR |
